# Sex Differences in Reaction to Chronic Unpredictable Stress in the House Mouse (*Mus musculus musculus*) of Wild Origin

**DOI:** 10.3390/biology15010054

**Published:** 2025-12-28

**Authors:** Tatiana Laktionova, Maria Klyuchnikova, Ilya Kvasha, Olga Laktionova, Vera Voznessenskaya

**Affiliations:** Severtsov Institute of Ecology and Evolution, Russian Academy of Sciences, 33 Leninski Prospect, 119071 Moscow, Russia; tatita.laktionova@sev-in.ru (T.L.); m_klyuchnikova@sev-in.ru (M.K.); igkvasha@sev-in.ru (I.K.);

**Keywords:** house mouse, sex differences, chronic unpredictable stress, corticosterone, behavior

## Abstract

Sex differences in stress response continue to be understudied in basic physiological and behavioral research. The current study aimed to investigate the sex-specific effects of chronic stress in wild-derived house mice subjected to chronic unpredictable stress (CUS). The use of wild-derived mice enhanced the ecological validity of our stress model. We applied CUS for 5 weeks based on protocols previously established in laboratory mice, with regular weighting and welfare checks. Control mice were not subjected to stress. After the 5-week exposure, behavioral tests were performed, blood and hair samples were collected for corticosterone measurement, and stress-sensitive organ weights were assessed. Stressed females, but not stressed males, gained significantly less body weight over the entire CUS period. After CUS, mice tended to have higher adrenal and thymus weights. In stressed females, we observed significantly prolonged grooming time in the open field test and fewer immobility episodes in the tail suspension test (TST). Stressed males displayed significantly shorter immobility time in TST. Stressed males, but not stressed females, had significantly higher levels of hair corticosterone, with a similar tendency in plasma. Our results indicate different CUS coping strategies in males and females and raise a question about the development of different protocols for the assessment of stress responses in males and females.

## Simple Summary

Did you know that male and female mice deal with long-term stress in different ways? It is a crucial question that has often been overlooked in science. To find out, we studied wild house mice, and their stress responses are closer to animals in nature. We exposed them to unpredictable stress for five weeks, then compared them to an undisturbed control group. The differences were pronounced. The stressed females gained much less weight than their unstressed counterparts did. Both sexes tended to have heavier stress-related organs, but their behavioral coping strategies diverged. Stressed females showed a conflict between exploring and avoiding risk. Stressed males, however, became more active in resisting inescapable stress. Male mice also produced higher levels of stress hormones. Our work reveals a key insight: There is no single stress response; instead, male and female mice have unique biological and behavioral ways for coping with pressure. These findings argue that to truly understand stress—in mice and, potentially, in humans—we must always consider sex-specific strategies. This means we may need to develop different ways to measure and treat stress for males and females.

## 1. Introduction

The notion that females present a greater behavioral and physiological variation led to a bias in animal research favoring an inclusion of male rodents [1]. As a result, sex differences in basic research of behavior and physiology in general and in stress responses in particular still remain understudied [2,3,4]. Another problem lies in the practice of treating males as the standard. This has shaped tests and parameters often applied, leaving the possibility of diverged results for females largely unexamined [1]. Thus, widely used protocols do not account for critical sex differences in behavioral and physiological responses. Notably, female rodents exhibit higher basal and stress-induced glucocorticoid secretion with greater variability than males [5]. Human studies also confirm this narrative, showing that women are more prone to stress-related disorders, such as anxiety disorders and depression [6,7,8].

In order to survive and thrive in a constantly changing environment, whether physical or social, animals must adapt their behavior, physiology, and morphology to the new conditions. They must be able to deal with both predictable changes, like seasonal variations and light cycles, and unpredictable events, like storms, predators, and diseases [9]. The stress response system is essential for the natural adaptability of mammals. The hypothalamic–pituitary–adrenal (HPA) axis is a neuroendocrine pathway that releases hormones, mainly glucocorticoids, in response to stressors. These hormones play a crucial role in orchestrating the stress response [10]. HPA activation is largely governed by higher-order brain circuits. Prefrontal cortex and basolateral amygdala play critical and often opposing roles in regulating this axis. The prefrontal cortex exerts top-down inhibitory control over HPA activity, while the basolateral amygdala is a key integrative hub that promotes anticipatory stress responses [11,12].

The characteristics of stress responses, namely duration and intensity, vary both among and within populations and individuals [13,14,15]. These variations are influenced both by parameters of external stressors, i.e., their type and temporal patterns, and by internal factors, i.e., sex, health condition, and life stage [5,16,17].

When faced with acute stress, the organism rapidly activates a cascade of physiological changes designed to optimize survival—a primal reaction known as the “*fight-or-flight*” response. This adaptive mechanism sharpens focus, mobilizes energy, and prepares the individual to either confront the threat or escape from it, thereby increasing their immediate chances of survival. However, a prolonged exposure to chronic stress can lead to immunosuppression and thus increased risk of disease. It may, in turn, lead to a decrease in the reproductive success and survival of individuals [18,19,20,21,22], ultimately negatively affecting the dynamics of the population as a whole [23,24].

The *Mus musculus (M. m.) musculus* is a common synanthropic species in both urban and rural areas of Russia. Both historically and in modern times, such coexistence is not favorable for the human population. *M. m. musculus* causes enormous economic damage and is associated with increased epidemiological risk. The area of *M. m. musculus* extends from Eastern Europe to Japan across Russia and Northern China [25]. A number of researchers refer the *M. m. musculus* subspecies to the rank of a separate species [26,27,28]. The majority of laboratory mouse strains are genetic mosaics of several subspecies of *M. m.* However, almost all of them are more closely related to another natural subspecies—*M. m. domesticus* [29]. Comparative studies have revealed significant behavioral and neuroendocrine divergence between wild-derived mice and laboratory inbred strains, including distinct exploratory patterns, social interaction profiles, and stress hormone responses [30,31,32,33,34]. These fundamental differences challenge the ecological validity of translating laboratory-based stress response findings to natural populations.

In the present study, we applied a commonly used protocol of chronic unpredictable stress (CUS) to the synanthropic house mouse *M. m. musculus* of wild origin. CUS involves random, intermittent, and unpredictable exposure to different stressors over several weeks [35]. To assess the effects of CUS, we chose to use non-invasive monitoring of hair corticosterone concentrations, which was adapted to the hair of wild-derived *M. m. musculus*. Hair corticosterone concentrations in animals, whether kept in captivity or living in the wild, can serve as a useful indicator of both individual and population well-being. Degradation of natural habitats can lead to elevated hair glucocorticoid levels in local rodents [36]. In another study, corticosterone levels in males (*M. m. domesticus*) increased with the extent of visible injuries or scars when living in semi-natural conditions, and it may reflect the metabolic costs of fighting for reproduction [37,38]. In addition to its cost-effectiveness, this method also allows for easy sample collection and long-term storage at room temperature [23].

This study aimed to examine sex-based divergence in chronic stress coping strategies in *M. m. musculus* of wild origin, using CUS as an experimental paradigm. Beyond its fundamental insights, this research holds direct promise for applied conservation science. The sex-specific stress signatures identified through the CUS paradigm—which effectively simulates the persistent pressures of human-dominated landscapes—can be translated into diagnostic toolkits. Such tools are urgently needed to monitor the physiological well-being of wild populations of small mammals, providing a basis for assessing ecosystem health and guiding targeted management strategies.

## 2. Materials and Methods

### 2.1. Animals

A total of 52 *M. m. musculus* (26 males and 26 females), 9 months old were used in this study. Wild-derived mice could live 2–3 years in captivity [39], and 9-month-old mice are classified within the adult-to-middle-aged transition cohort and are well within the norm for experimental studies in this model. Furthermore, this age was selected to ensure animal welfare: unlike aged inbred strains (e.g., C57BL/6), which can become obese, wild-derived mice remain lean but acquire sufficient body mass to tolerate metabolic stressors (e.g., food and water deprivation or exposure to cold) without immediate health risks, which was a critical consideration for our chronic stress paradigm. Females were randomly cycling. Test subjects were second- and third-generation laboratory bred offspring of 11 parental pairs trapped from the wild in Vladimir Oblast, Russia (coordinates: 56°21′ N 41°21′ E). This location is far from hybridization zones and potential sites of invasion of other subspecies. To mitigate inbreeding, we avoided pairing animals captured within 2 km of each other. Our rationale is that two or three generations of laboratory breeding are too short a time for significant artificial selection to act. As such, these mice retain the essential genetic architecture and latent phenotypic potential of their wild ancestors [40]. Animals were housed individually in opaque plastic cages under standard vivarium conditions with separate air ventilation. Mice were acclimated to single housing for 10 days before the experiment. The room temperature was maintained at 21 ± 2 °C, and the lighting conditions were 14 h of light and 10 h of darkness, with lights on at 8 a.m. A 14-h light/10-h dark period was employed to mimic the extended daylight conditions of temperate-latitude summers. This cycle is frequently used in ecological and circadian research utilizing wild-derived mouse populations [41]. Pelleted food, oat grains, sunflower seeds (Laboratorkorm, Moscow, Russia), and water were provided ad libitum. To enrich the environment, we provided deep bedding and nesting material and scattered natural foods to encourage natural foraging instincts. Experiments were conducted in compliance with the EU Directive 2010/63/EU and local regulations. This study was reported in accordance with the ARRIVE guidelines (Table S9 of the Supplementary Materials). All procedures were approved by the Bioethics Committee of the Severtsov Institute of Ecology and Evolution. Every effort was made to minimize the number of animals used and their suffering.

### 2.2. Experimental Design

The animals were randomly assigned to two groups: a CUS group and a control group. To control for litter effects and genetic variability, progeny from each breeding pair were redistributed across the groups. The experimental schedule and group sizes are shown in Table 1. Group sizes were decided based on previous behavioral research in wild-derived mice (e.g., [34,42]). The control group of males (*n* = 14) was slightly larger than the stress group (*n* = 12). This design change was implemented for two primary reasons: (1) to take full advantage of animals already available from our breeding colony; (2) to increase the statistical power and precision of our baseline measurements. Over 36 consecutive days (5 weeks), the CUS group mice were exposed daily to various stressors, while the control group mice remained unstressed, undergoing only body weight and welfare assessments. Body weight was measured once every 1–2 weeks. Animals were distributed into four balanced cohorts. After completion of the CUS procedures and one to four days of rest (duration varied systematically by cohort), behavioral tests were performed as listed in Table 1. Following this, hair samples were collected and terminal procedures were performed by trained personnel. Trunk blood was drawn and target organs were dissected for weighing after humane killing. All samples and video recordings were assigned numerical codes to facilitate the blinded analysis. Our data collection protocol was a deliberate and necessary consequence of our animal model. Since (1) wild-derived mice exhibit a higher stress reactivity (e.g., they are more sensitive to handling and laboratory manipulations) than classical laboratory strains [43], and (2) this study is the first to subject *M. m. musculus* to a CUS, it was paramount to avoid confounding variables. The confounding effects of additional manipulations could have masked or overwhelmed the specific impact of the core CUS paradigm.

### 2.3. Chronic Unpredictable Stress (CUS)

The procedures for CUS were adapted from protocols previously established on laboratory mice [44,45]. The following stressors were included in the protocol: physical restraint in 50 mL conical tubes for one hour, wet bedding for twelve hours, cage tilted at 45 degrees for one hour, lights on overnight, cold chamber at 4 °C for one hour, water or food deprivation for sixteen hours, hot air stream for 15 min, exposure to cat urine odor (100 μL), or exposure to rats (*Rattus norvegicus*) for one hour. Fresh cat urine was collected from an adult unneutered female cat and stored at −40 °C until use. For exposure to rats, cages with mice were moved to the room where the laboratory rat colony was maintained. The duration of food and water deprivation was shortened due to the smaller weight of wild-derived mice compared to mice of laboratory strains, a necessary adjustment guided by pilot data. A single stimulation pattern was applied daily, except one day when two patterns were applied. Stress exposures started at different times between 8 a.m. and 9 p.m., and the sequence of stressors varied each day. The goal of the CUS approach is to reduce possible habituation to the stressor, which can develop if the same stimulus is presented repeatedly. More information on our CUS protocol is provided in Table S6 of the Supplementary Materials.

### 2.4. Behavioral Tests

#### 2.4.1. Open Field Test (OFT)

We used one of the modifications of a standard OFT—“hole board”. The hole board apparatus is a non-transparent plastic cylinder measuring 150 cm in diameter with walls 120 cm high. The arena was raised 3 cm above the ground on a plastic stand. The floor was divided into three zones: the central zone, the buffer zone, and the wall zone. There were eight cone-shaped holes (3 mm in diameter) in the floor, evenly spaced for each zone, and one hole in the center. At the start of the test, each mouse was placed in the wall zone of the arena.

During a 60-min trial, we simultaneously obtained behavioral annotations and digital video recordings using equipment mounted above the open arena (Sony 960H CCD Effio 700TVL Security Mini CCTV Camera, Tokyo, Japan). The following behavioral patterns were recorded: number of central zone entries, number of rearings, hole pokes, total duration of grooming, total duration of immobility, and number of defecation boluses. The number of rearings (the mouse is stationary on its hindlimbs while raising its forelimbs off the ground and extending the body vertically) was considered as a measure of vertical investigatory activity. The number of holes investigated served as a characteristic of horizontal investigatory activity. The number of defecation boluses was used as a measure of emotionality. Total time of immobility (“freezing” behavior) was used as a correlation of passive avoidance behavior. Total time of grooming was used as an indicator of a conflict between tendency to exploration and passive avoidance behavior [46,47]. Total distance traveled and time spent in the predefined zones were quantified using ToxTrac v025.1.0 software [48]. The arena was cleaned with 70% ethanol after each animal to eliminate olfactory cues.

#### 2.4.2. Tail Suspension Test (TST)

TST was conducted in accordance with methods described by Steru et al. [49] and Cryan et al. [50], with some minor modifications. Each mouse was suspended by attaching a piece of tape approximately 1 cm from the tip of its tail, ensuring that its forelimbs were at least 10 cm above the floor of the apparatus. To prevent the mouse from climbing the tail, the tail was passed through a small tube. The latency to the first immobile episode, duration of immobile episodes, and number of episodes were quantified using a digital video recording (Full HD Panasonic HC-V160 camera, Petaling Jaya, Selangor, Malaysia) and subsequent analysis. Testing time was extended to 10 min given the superior physical abilities of wild-derived mice compared to laboratory-bred mice [42,43,51].

### 2.5. Hair, Blood, and Organ Samples Collection

Hair collection was performed after the completion of behavioral tests and one day before the mice were sacrificed (Table 1). Hair from the lower back of the mouse (~25 mg) was shaved using an electric razor as close to the skin as possible without causing skin trauma. Shaved hair samples were then stored in aluminum foil bags at −4 °C, away from light, until steroid extraction and analysis.

Blood was collected from the trunk after decapitation and placed in micro-sample lithium heparin tubes (Sarstedt, Nümbrecht, Germany). Plasma samples were obtained after 10 min of centrifugation at 2000 g and 20 °C and stored at −40 °C until analysis.

The adrenal glands and thymus were removed and weighed immediately after humane killing.

### 2.6. Corticosterone Measurement

The concentration of corticosterone, the main glucocorticoid in the house mouse, was measured using the enzyme-linked immunosorbent assay (ELISA) in both plasma and hair samples.

Hair corticosterone extraction and analysis were performed according to previously described protocols, with minor modifications [52,53]. Hair samples were washed twice for 5 min with 3 mL of pure isopropanol (CAS 67-63-0, EKOS-1, Moscow, Russia) on a horizontal shaker to remove external contaminants that may contain steroids. After washing, excess isopropanol was removed, and the samples were allowed to dry overnight under a fume hood at room temperature. The dried hair samples were homogenized into a fine powder using a TissureLyser II ball mill (Qiagen, Hilden, Germany) with stainless-steel grinding jars for 5 min at 25 Hz. Subsequently, 20 mg of powdered hair sample was weighed. The duration and the frequency of grinding were selected empirically on the basis of the physical characteristics of the sample (fine grinding into powder but not mush), as well as the previous results of the corticosterone measurement. The powdered samples were incubated on a horizontal shaker for 24 h with 1 mL of 80% methanol (HPLC grade from Component-reactiv LLC, Moscow, Russia). After centrifugation (2 min at 12,000 rpm), the supernatant was transferred to a clean microtube and diluted with distilled water to achieve a methanol concentration of 55%. Corticosterone concentrations in methanol extracts and blood plasma were measured using an ELISA kit (#K210R, Chema, Ltd., Moscow, Russia). The antibodies in the kit had cross-reactivity with other steroids of 1.6% with cortisol, 2.2% with progesterone, and less than 0.1% for all other steroid hormones tested. The sensitivity of the kit is 5.0 nmol/L, and the coefficient of variation (CV) is <8%. The absorbance was measured at a wavelength of 450 nm using a SpectraMax 340PC 384 spectrophotometer (Molecular Devices, Silicon Valley, CA, USA). Data analysis was performed using SoftMax Pro Software (version 7.0, http://www.moleculardevices.com/pages/software/softmax.html, accessed on 20 November 2025).

### 2.7. Statistical Analyses

Results are reported as the mean ± standard deviation (SD) or median (Q1–Q3). Prior to applying any analyses, the data were visually inspected using Q–Q plots, and boxplots were checked as well to fit in a normal distribution (Shapiro–Wilk test, *p* > 0.05) and to be homoscedastic (Levene’s test, *p* > 0.05). Differences between groups were analyzed using two-way analysis of variance (ANOVA), with group (CUS or control) and sex as the between-subjects variables. Pairwise comparisons of group means were performed using the *t*-test for a list of contrasts specified as follows: control males vs. control females, stressed males vs. stressed females, control males vs. stressed males, and control females vs. stressed females. As these comparisons were planned a priori and constitute a limited set of hypotheses, *p*-values are reported without adjustment to maximize the statistical power for these specific questions [54,55]. Nevertheless, for full transparency, the corresponding *p*-values adjusted for multiple comparisons using the Holm–Bonferroni method are also provided in the Supplementary Tables. If any severe violations of ANOVA assumptions were noticed, non-parametric Mann–Whitney tests were used to check the statistical significance of differences between the same group pairs as listed above. Sex-specific Spearman correlation analyses for the selected variables (Figure S1 and Table S7) were performed under the assumption that correlation patterns would be consistent across treatment groups within each sex. A multivariate analysis of variance (MANOVA) was additionally conducted with exploratory aims on a subset of six variables and is presented in the Supplementary Materials (Table S8) due to its post hoc nature and sample size limitations. An alpha level of 0.05 was used as a significance criterion for all statistical tests. All statistical analyses were performed using the *rstatix* package (version: 0.7.2, https://CRAN.R-project.org/package=rstatix, accessed on 1 March 2023) in RStudio (version 2024.04.2+764).

## 3. Results

### 3.1. Body Weight Change

**Figure 1:**
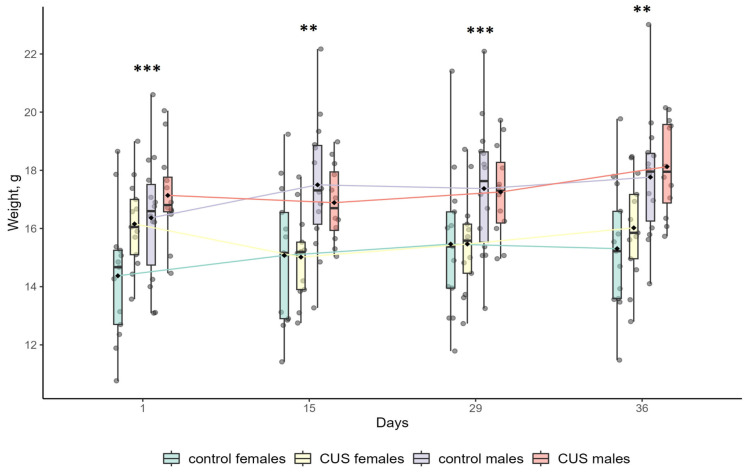
Body weight changes in mice. Asterisks indicate the significance level of sex differences in body weight of mice revealed by two-way ANOVA with sex and group as the main effects. ** *p* < 0.01 and *** *p* < 0.001. At Day 1, there was a significant effect of the group (*p* < 0.05). No other significant effects or their interactions were found. Full ANOVA results are available in Table S1 of the Supplementary Materials. Boxplot elements: The central line represents the median, the box boundaries are the first and third quartiles, the whiskers extend to the minimum and maximum values, and the rhombus is a marker of the mean. Raw data are shown as points. *n* = 12–14 per group.

In our study, two-way ANOVA and the *t*-test were performed to analyze the effects of CUS exposure and sex on body weight and body weight gain during the experiment. The results are shown in Figure 1 and Table 2.

At baseline (Day 1), female mice had a significantly lower body weight than males, (0.9-fold; 15.26 ± 2.08 g vs. 16.72 ± 1.92 g; ANOVA, *p* < 0.01; effect size, η^2^ = 0.143; *n* = 26 and *n* = 26, respectively). Full ANOVA results are shown in Table S1 of the Supplementary Materials. This pattern remained unchanged throughout the experiment (Figure 1). The dynamics of body weight gain are shown in Table 2. For the entire period of experimental stress (Days 1–36), there were significant main effects of sex (ANOVA, *p* < 0.05; males > females; effect size, η^2^ = 0.135) and group (ANOVA, *p* < 0.01; stress < control; effect size, η^2^ = 0.123) on body weight gain, and interaction between the effects was not significant (ANOVA, *p* > 0.1; Table 2). Females exposed to CUS lost, on average, 0.13 g (−1%), while control females gained 0.93 g (+7%) of body weight during the entire study period. This difference was statistically significant (*t*-test, *p* < 0.01; effect size, Cohen’s d = 1.32; Table 2). In contrast, males showed no significant difference in body weight gain between the CUS and control groups over the entire study period (*t*-test, *p* > 0.1; Table 2). Both groups exhibited comparable weight gains: 0.99 g (+6%) and 1.40 g (+9%), respectively (Table 2). However, the impact of stress on body weight gain was not uniform across the experimental timeline. Over the first two weeks of the study (Days 1–15), CUS significantly suppressed body weight gain in both male and female mice (*t*-test, *p* < 0.01 and *p* < 0.001, respectively; effect size Cohen’s d = 1.38 and d = 2.79, respectively; Table 2). However, during the following two weeks (Days 15–29), there was no significant effect of stress on body weight gain (ANOVA, *p* > 0.1; Table 2). During the last week of exposure to stressors (Days 29–36), the body weight gain of stressed males did not differ significantly from that of the control males, whereas females exposed to CUS showed a significantly greater weight gain than their control counterparts (*t*-test, *p* > 0.1 and *p* < 0.05, respectively; effect size Cohen’s d = −0.985 in females; Table 2).

Analyses assessing correlations separately by sex showed no evidence of a differential relationship between initial body weight and subsequent weight change; the association was non-significant in both groups (Figure S1 and Table S7).

### 3.2. Post-Mortem Stress-Sensitive Organ Weights

**Figure 2:**
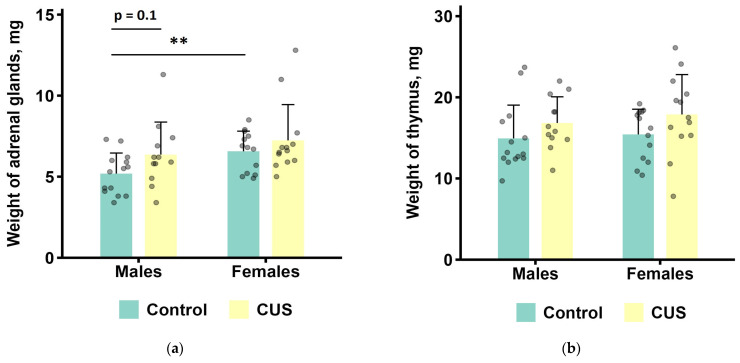
Post-mortem organ weights after CUS exposure in *M. m. musculus*: (**a**) adrenal glands; (**b**) thymus. Bars are the mean values, bar errors show SDs. ** *p* < 0.01, *t*-test, *n* = 12–14 per group. ANOVA results are presented in Table 3. Raw data are shown as points. Full *t*-test results are available in Table S3 of the Supplementary Materials.

Two-way ANOVAs were performed to determine the effects of CUS exposure and sex on adrenal glands and thymus weights (Figure 2 and Table 3). CUS induced a marginally significant increase in adrenal gland weight (1.2-fold; ANOVA, *p* = 0.059; effect size, η^2^ = 0.072). The average weight of adrenal gland in females was significantly higher than in males (1.2-fold; ANOVA, *p* < 0.05; effect size, η^2^ = 0.106). Furthermore, there was a marginally significant main effect of the stress on thymus weight (ANOVA, *p* = 0.051; effect size, η^2^ = 0.077), with greater average organ weight in the CUS group compared to the control (1.2-fold). All the other tested effects and their interactions were not significant (ANOVA, all *p* values > 0.1, Table S3 of the Supplementary Materials).

Sex-specific correlation analyses revealed differential associations between adrenal glands weight and thymus weight (Figure S1 and Table S7). While females showed no significant correlation between these variables (Spearman’s ρ = 0.25, *p* = 0.22), males demonstrated a moderate positive association (Spearman’s ρ = 0.46, *p* = 0.018).

### 3.3. Behavioral Changes

#### 3.3.1. OFT

**Figure 3:**
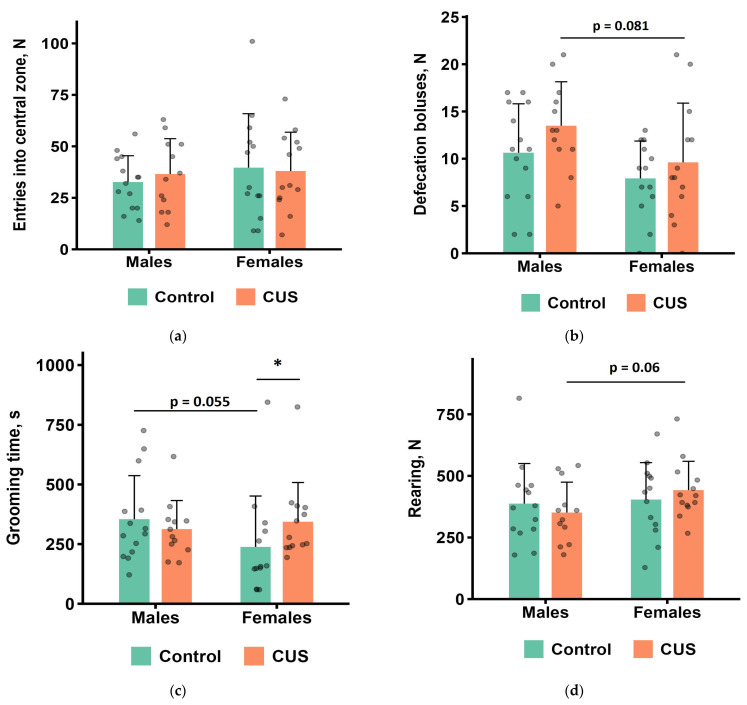

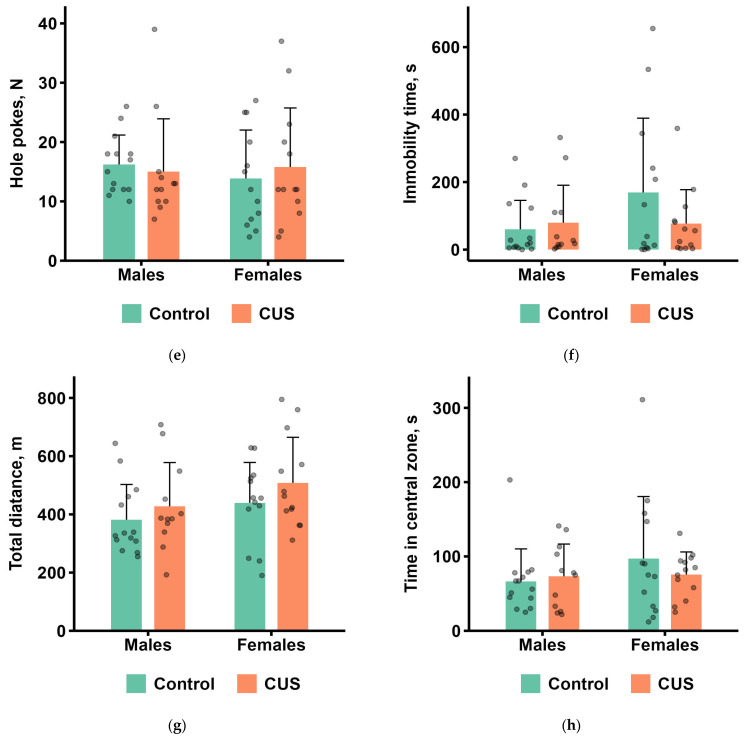
OFT parameters after CUS exposure in *M. m. musculus*: (**a**) the number of entries to the central zone of the arena; (**b**) the number of defecation boluses; (**c**) total grooming time; (**d**) the number of rearings; (**e**) the number of hole pokes; (**f**) total immobility time; (**g**) total distance travelled; (**h**) time in the central zone of the arena. Bars are mean values, and bar errors show SDs. * *p* < 0.05, Mann–Whitney test, *n* = 12–14 per group. Raw data are shown as points. Full Mann–Whitney test results are available in Table S4 of the Supplementary Materials.

The results of the OFT are shown in Figure 3a–h. In males, no significant differences were found between the control and CUS groups for any of the measured parameters during 60 min of observations after the completion of the 5-week CUS protocol (Mann–Whitney test, all *p* values > 0.1; Figure 3a–h). In females, the CUS group showed significantly longer grooming in the wall zone of the arena (Mann–Whitney test, *p* < 0.05, effect size = 0.417; Figure 3c). CUS males tended to produce more fecal boluses and fewer rearings compared to CUS females, whereas control males tended to spend more time on grooming than control females (Figure 3b–d).

Examination of the OFT parameters through Spearman correlation analysis demonstrated multiple significant correlations (Figure S1 and Table S7). Inverse correlation patterns emerged between sexes for defecation boluses vs. immobility time. Males showed a positive correlation (ρ = 0.43, *p* = 0.027), while females demonstrated a negative correlation of similar magnitude (ρ = −0.50, *p* = 0.0094).

#### 3.3.2. TST

**Figure 4:**
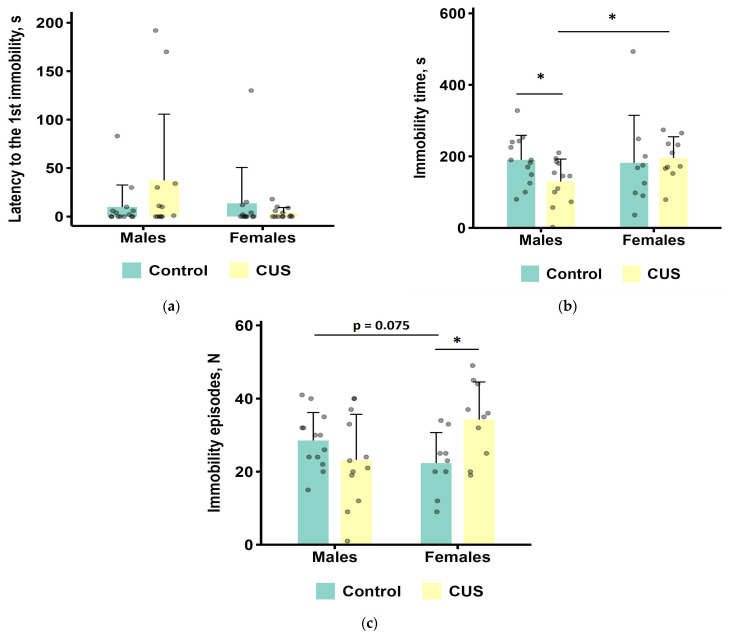
TST parameters after CUS exposure in *M. m. musculus*: (**a**) latency to the first immobility episode; (**b**) the total duration of immobility; (**c**) the number of immobility episodes. Bars are mean values, and bar errors show SDs. * *p* < 0.05, Mann–Whitney tests, *n* = 9–14 per group. Raw data are shown as points. Full Mann–Whitney test results are available in Table S4 of the Supplementary Materials.

The results of the TST are shown in Figure 4a–c. The data from one male control, three female controls, and three stressed females were excluded from the analysis of immobility time due to their tail climbing behavior.

In females, CUS exposure caused a significant increase in the number of immobility episodes compared to the control group (Mann–Whitney test, *p* < 0.05; effect size = 0.508; Figure 4c). However, the total immobility time and latency to the first immobility episode did not change significantly (Mann–Whitney test, all *p* values > 0.1; Figure 4a,b).

In males, CUS exposure resulted in a significant reduction in the total time spent immobile (0.68-fold on average, Mann–Whitney test, *p* < 0.05 effect size = 0.408; Figure 4b). However, the latency to the first episode of immobility was not significantly affected (Mann–Whitney test, *p* > 0.1, Figure 4a).

CUS females had a significantly longer time of immobility and tended to have a higher number of immobility episodes compared to CUS males (Mann–Whitney test, *p* < 0.05 and *p* = 0.075, respectively; effect sizes, 0.471 and 0.387, respectively; Figure 4b,c).

The TST parameters showed intercorrelated patterns (Figure S1 and Table S7). Males demonstrated a strong positive correlation between total immobility time and immobility episode count (ρ = 0.70, *p* < 0.001) and a moderately negative correlation between immobility latency and immobility time (ρ = −0.51, *p* = 0.0089). Parallel correlation structures were identified in females.

### 3.4. Hair and Plasma Corticosterone

All corticosterone data (plasma and hair) were analyzed using Mann–Whitney *U* tests. While hair corticosterone data did not show significant deviation from normality (Shapiro–Wilk *p* ≥ 0.05 in all groups), this approach was adopted for consistency due to the non-normal distribution of plasma data. Data from three male controls, one CUS male, two female controls, and one CUS female mice were excluded from the analysis of blood plasma corticosterone due to hemolysis or small sample volume. We found a significantly higher level of plasma corticosterone in control females than in control males (*p* = 0.028, *n* = 11 per group). There was also a trend towards higher hormone levels in CUS males compared to control males (*p* = 0.088, *n* = 11 per group). No other statistically significant differences were found (Table S5 in the Supplementary Materials). For the combined groups of mice, there were no statistically significant differences between the CUS and the control groups (Mann–Whitney test, *p* = 0.264), but there was a tendency for higher corticosterone level in females compared to males (Mann–Whitney test, *p* = 0.0544, Table S5 in the Supplementary Materials).

**Figure 5:**
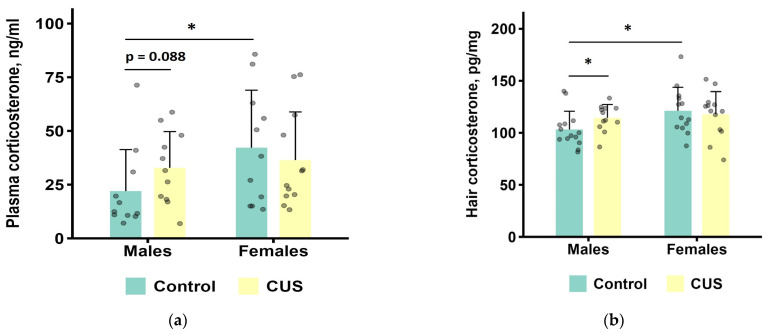
Corticosterone levels after CUS exposure in *M. m. musculus*: (**a**) in blood plasma. *n* = 11–12 per group; (**b**) in hair. *n* = 12–14 per group. Bars are mean values, and bar errors show SDs. Solid lines indicate significant differences and tendencies: *—*p* < 0.05, Mann–Whitney test. Raw data are shown as points. Full results of the statistical analysis are available in Table S5 of the Supplementary Materials.

For corticosterone concentrations in hair (Figure 5b), a Mann–Whitney test showed that CUS males had significantly higher levels of corticosterone than control males (*p* = 0.036, *n* = 12–14 per group). In addition, control males had lower hair corticosterone than control females (*p* = 0.022, *n* = 13–14 per group), while no other statistically significant differences were found (all *p* values > 0.1) (Table S5 of the Supplementary Materials). For the combined groups of mice, there were no statistically significant differences between the CUS and the control groups (Mann–Whitney test, *p* = 0.259), but females had higher corticosterone level as compared to males (Mann–Whitney test, *p* = 0.0406, Table S5 in the Supplementary Materials).

No significant correlation was detected between blood plasma and hair corticosterone levels in either sex (males: ρ = 0.16, *p* = 0.485; females: ρ = 0.046, *p* = 0.834), as shown in Figure S1 and Table S7.

## 4. Discussion

To the best of our knowledge, we are the first to examine the effects of chronic stress exposure in the house mouse of wild origin under controlled laboratory conditions. We have used the CUS protocol, a well-established model for inducing behavioral, hormonal, and immunological changes associated with chronic stress in laboratory strains of mice [45,56,57]. The CUS procedure lasted 5 weeks, and a single stressor from a pool of ten types of stressors was applied each day in an alternating regime to reduce the process of habituation, which commonly develops with repeated exposure to homotypic stimuli [58]. To focus on the sex-specific effects of chronic stress in an ecologically relevant animal model, we used both male and female *M. m. musculus* of wild origin as test subjects. Sex-matched, undisturbed control groups were included for comparison. The major findings of our study were as follows. For the first two weeks of CUS, body weight gain was significantly lower in stressed mice as compared to controls, and this was evident in mice of both sexes. However, stressed females, but not stressed males, gained significantly less body weight than the same-sex control groups over the entire CUS period of 5 weeks (1). Compared to controls, stressed mice tended to have higher post-mortem adrenal and thymus weights (2). Behavioral testing after 5 weeks of CUS revealed several significant sex-specific effects of stress, such as prolonged grooming time in females in OFT, fewer immobility episodes in females in TST, and shorter immobility time in males in TST; however, the majority of assessed parameters remained unaffected (3). Stressed males, but not stressed females, had significantly higher levels of corticosterone in hair, with a similar tendency in plasma, as compared to the same-sex controls (4). We have to note that our main findings (3) and (4) derive from analyses without multiplicity corrections, an approach deemed suitable given the exploratory focus of the study and planned comparisons. Separate correlation analyses for each sex uncovered distinct patterns of association between the variables.

Over the 5-week study duration, we monitored changes in body weight of mice in response to CUS application. Males weighed significantly more than females, and it is in line with the sexual size dimorphism (males larger than females) previously reported in *M. m. musculus* from different populations [59,60]. Chronic stress commonly leads to a reduction in body weight and/or slower weight gain in laboratory animal models [56,61,62], and similar outcomes were reported in wild rodents [63,64]. However, in some cases, stress can have the opposite effect, leading to increased food consumption and weight gain, e.g., depending on the social status and strain of the animal or its diet [65,66,67]. The results of our study show that chronically stressed mice of wild origin, both males and females, gained significantly less weight for the first two weeks than the controls, thereby proving the efficacy of the applied CUS procedure. Further development of the stress effect on body weight gain over time appeared to be sex-specific. The reduction in body weight gain under stress conditions was more pronounced in females than in males, as concluded from the gains for the entire period of CUS. Nonetheless, the significant reversal of the inhibitory effect of stress on body weight gain was observed on days 15–29 in males and days 29–36 in females. These changes may be attributed to either adaptation to stress or to dysregulation of feeding habits and metabolism caused by stress.

Prolonged exposure to stress can lead to hypertrophy or atrophy of select organ systems [68,69,70]. Among the most sensitive characteristics of chronic stress are adrenal and thymus weights. The expected stress-related changes in organ weights are an increase for the adrenal glands and a decrease for the thymus. Accordingly, we detected a tendency in the chronically stressed mice to have higher adrenal glands weights as compared to the controls. In addition, significantly greater adrenal gland weights were found in females compared to males, and that is consistent with many previous research observations on sexual dimorphism of adrenal gland development [71]. We also observed a tendency for elevated thymus weights in mice subjected to CUS, which is not in line with a classical stress-induced thymic involution [69]. The latter effect may be related to ongoing adaptation processes, and regenerative thymic hyperplasia has previously been reported in mice following a reduction in stress load [72].

Activation of the stress response system frequently promotes behavioral alterations involved in regulating homeostasis. Specifically, chronic stress leads to hyperactivity of the basolateral amygdala and dysfunction of the prefrontal cortex, which impairs HPA feedback, resulting in maladaptive behavioral and cognitive outcomes, including anxiety and memory deficits [11,12]. In animal models, changes in emotion-related behaviors were previously reported in response to stress, such as induced anxiety, defensive reactions, decreased social interactions, and unusual sexual behavior [73]. We evaluated behavioral responses after completion of the CUS procedure and the subsequent short period of rest (1–4 days) to avoid measuring immediate reactions to the applied stressors. We utilized the OFT to investigate changes in anxiety/boldness, exploration, and locomotion, since similar settings are commonly used to assess behavioral parameters of wild rodent species under semi-field or laboratory conditions [74,75]. Regarding the TST used in our study, to the best of our knowledge, this test has not been evaluated in animals of wild origin to date. TST is analogous to the forced swim test (FST, also known as Porsolt’s test) [76]. Previously, we demonstrated that *M. m. musculus* of both sexes displayed episodes of immobility (“behavioral despair”) in FST similar to laboratory mice [42]. The advantages of TST over FST include absence of test-induced hypothermia and no need for post-treatment [77]. Like FST, TST can be viewed as an inescapable stressor challenge situation in which the relationship between the two types of animal coping strategies, active and passive, can be assessed [78]. The magnitude of the stress load for the animals in this test is much greater than that experienced in the OFT.

In our study, under low levels of stress experienced during the OFT, no alterations in behavior of chronically stressed male mice were observed, while chronically stressed females exhibited significantly increased grooming time relative to controls. Parameters of grooming behavior are considered to be sensitive to stress. The potential stress effects on the grooming behavior include a decrease or, conversely, an increase in this activity. The intensity of the stress is likely to be a determining factor in this regard [79]. The prolonged grooming time can be attributed to development of a conflict between active exploration and passive avoidance behavior [44,79]. Consistently, in our previous study, wild-derived *M. m. musculus* exhibited significantly increased grooming during 10-min OFT following three daily 1-h applications of restraint stress [42].

More prominent differences between the control and CUS groups were observed in mice of both sexes under the high-stress conditions of TST. Males exposed to CUS had significantly shorter total immobility time relative to the control group, which is indicative of the induction of an active coping strategy. Whether this result should be interpreted as an adaptation to CUS or a specific stress response remains the question, as effects of genetic background have been previously reported for this test in laboratory strains. For instance, in one study, C57BL/6J males, unlike BALB/cJ males, had decreased immobility time in the TST after chronic unpredictable mild stress, whereas, in the FST, the expected increase in immobility was observed in both strains [80]. Stressed females in our study had a significantly greater number of immobility episodes when compared to the control group. We assume that the latter parameter reflects “indecision” in choosing between active and passive coping strategies, similar to grooming in the OFT.

Overall, based on our observations of behavioral and morphophysiological alterations, females appeared to be more affected by exposure to CUS than males. We may suggest that CUS potentiates the manifestation of sex differences explained by sexual evolution theory, such as the enhancement of active coping in males but not in females. Duration of the effect is subject to further research, as the consequences of chronic stress can affect an animal’s behavior and physiological responses long after the stressor has ceased. Though the CUS procedure was introduced to overcome the shortcomings of chronic stress models related to adjustment to treatment, adaptation may still occur [81]. In our study, males seemed to adapt to CUS faster than females.

In the present study, we have assessed corticosterone levels in plasma and hair of *M. m. musculus* after the end of CUS exposure. The content of glucocorticoids in various biological matrices (blood, urine, feces, and hair) reflects the different periods of activity of the HPA axis. The concentration of glucocorticoids in blood rises within a few minutes in response to acute stressors. Stress-induced glucocorticoid changes in urine and feces are detected within a few days [23,82]. The concentration of glucocorticoids in hair is mainly determined by the overall activity of the HPA axis during the entire period of hair growth [23] and thus may serve as a better indicator for chronic stress than plasma corticosterone. Since it is almost impossible to avoid additional stress during blood sampling in wild animals, plasma corticosterone may be more related to an acute stress response to the procedure itself rather than to CUS. Our finding of non-significant correlations between plasma and hair corticosterone levels confirms their dissociation and validates the distinct temporal resolutions of these two measures. Additionally, we have to acknowledge that the phase of the estrous cycle in randomly cycling females could potentially contribute to variability in plasma corticosterone but not to variability to hair corticosterone. In general, an increase in basal plasma corticosterone level and a greater accumulation of corticosterone in hair are expected after chronic stress in rodents [53,83,84,85]. However, over time, chronically altered HPA axis function may lead to exhaustion of the existent hormonal pool and, thus, to glucocorticoid deficiency [4,62].

In our study, the effect of stress on hair or plasma corticosterone levels was not detected for the entire study sample. However, higher hormone levels were detected in plasma and hair of stressed males as compared to controls (*p* = 0.088 and *p* = 0.036, respectively), but there was no similar tendency in females. As mentioned earlier, higher plasma corticosterone in CUS males as compared to male controls may be related not only to elevated basal levels of the hormone but also to increased stress reactivity of the test subjects. No change in corticosterone levels in stressed females as compared to female controls is an issue for further research, since, from our behavioral and morphophysiological observations, females were definitely affected by CUS. Previously, sex-related differences in changes of corticosterone after CUS, such as absence of an effect in female rodents, were reported by some researchers [62,86], though measurements in these studies were performed on plasma samples. Studies of the effect of chronic stress on hair corticosterone levels in female laboratory mice are scarce. Jarcho et al. [82] observed elevated hair corticosterone compared to baseline levels and controls in outbred female mice subjected to five weeks of social instability. Hohlbaum et al. [87] investigated long-term effects of housing conditions in female C57BL/6JRj mice and found no change in hair corticosterone levels; meanwhile, the separated pair housing conditions as compared to single housing led to some long-term behavioral changes that could be attributed to experienced stress.

Our study may serve for biological validation of the method of corticosterone measurement in hair for wild-derived mice. First, we observed a higher level of the hormone in chronically stressed males as compared to controls. Second, female *M. m. musculus* exhibited elevated corticosterone levels as compared to males. It was expected, as previous studies in rodents have shown that the level of glucocorticoid secretion is normally higher in females than in males and that there are sex differences in the variability of measured values [5,88].

Regarding the lack of differences in hormone levels between female mouse groups in our study, we can suggest that, during the first weeks of the CUS, corticosterone levels were elevated in accordance with the acute phase of stress, which is consistent with the reduction in body weight gain during this period. Then, this phase could be followed by a decrease in HPA activity, resulting in hair corticosterone concentrations similar to those of the control group by the 5th week of stress exposure. In a recent study, Colding-Jørgensen et al. [89] proposed and experimentally confirmed that glucocorticoids not only accumulate but also diffuse free along the hair shaft, so relatively quick elimination of corticosterone in the case of hormone depot depletion in organisms appears possible. Accordingly, Gáll et al. [90] observed reduced corticosterone accumulation in hair after 4 weeks of chronic unpredictable mild stress in male Wistar rats. Therefore, we suggest that the CUS protocol we have used should be modified to the type of stressors applied and/or their duration for females in order to better detect changes in HPA axis activity by measuring hair corticosterone. Including more ethologically relevant sex-specific social stressors may help to address this issue [4,62].

## 5. Conclusions

To the best of our knowledge, this study is the first to describe the effects of chronic stress exposure in house mice of wild origin under controlled laboratory conditions. Our findings may facilitate the interpretation of both field experiments and studies employing conventional laboratory mouse strains.

Our study confirms the importance of including females in research on chronic stress, as males and females of *M. m. musculus* showed different responses to the 5-week CUS. CUS affected physiology and behavior of *M. m. musculus* in a sex-dependent manner. In males, our results may reflect an activation of the HPA axis in the first weeks of the experiment and subsequent adaptation to stress at least by the end of the protocol. In the case of females, after the acute phase of stress, we can argue a suppression of HPA activity in later weeks of the protocol. Thus, the study revealed a sex-based divergence in chronic stress coping strategies in *M. m. musculus*: males showed active strategy, whereas females followed a rather passive one. Taking the latter into consideration, future studies should address the need for optimized stress protocols in females.

## Figures and Tables

**Table 1:** Experimental schedule.

Stage	Timeline (41–44 Days in Total)		Procedures	
			CUS Group 12 Males + 13 Females	Control Group 14 Males + 13 Females
experimental chronic stress	↓	36 days	CUS protocol	undisturbed
			body weight on Days 1, 15, 29, and 36	
rest	↓	1–4 days	undisturbed	
behavioral tests	↓	2 days	open field test (OFT) (on the 1st day), tail suspension test (TST) (on the 2nd day)	
hair collection	↓	1 day	hair sampling	
terminal	↓	1 day	humane killing, blood collection, organ weights	

CUS, chronic unpredictable stress. Arrows mean “continue”.

**Table 2:** Body weight gain.

Days	Control, Males	CUS, Males	Control, Females	CUS, Females	Two-Way ANOVA, *p*-Level			*t*-Test, *p*-Level
	*n* = 14	*n* = 12	*n* = 13	*n* = 13	sex	group	Sex * group	
1–15	1.14 ± 1.23 (+7%)	−0.25 ± 0.71 (−1%)	0.70 ± 0.76 (+5%)	−1.14 ± 0.55 (−7%)	** m > f	*** S < C	n.s.	Sf < Cf *** (d = −2.79) Sm < Cm ** (d = −1.38) Sm > Sf ** (d = 1.40) Cm > Cf n.s.
15–29	−0.13 ± 0.71 (−1%)	0.37 ± 0.48 (+3%)	0.39 ± 0.77 (+3%)	0.45 ± 0.45 (+2%)	# m < f	n.s.	n.s.	Sf > Cf n.s. Sm > Cm * (d = 0.820) Sm < Sf n.s. Cm < Cf #
29–36	0.39 ± 0.48 (+2%)	0.87± 0.98 (+4%)	−0.16 ± 0.72 (−1%)	0.56 ± 0.73 (+5%)	* m > f	** S > C	n.s.	Sf > Cf * (d = 0.985) Sm > Cm n.s. Sm > Sf n.s. Cm > Cf * (d = 0.896)
1–36 (Total)	1.40 ± 1.39 (+9%)	0.99 ± 0.94 (+6%)	0.93 ± 0.83 (+7%)	−0.13 ± 0.80 (−1%)	** m > f	* S < C	n.s.	Sf < Cf **(d = −1.32) Sm < Cm n.s. Sm > Sf **(d = 1.29) Cm > Cf n.s.

Significance levels: *** *p* < 0.001, ** *p* < 0.01, * *p* < 0.05, # *p* < 0.1, and n.s.—*p* > 0.1; m, males; f, females; S, CUS; C, control; d = Cohen’s d for the significant effects. Full statistical analyses results are available in Table S2 of the Supplementary Materials. Data are expressed as the mean ± SD, g.

**Table 3:** Post-mortem organ and body weights.

	Control, Males	CUS, Males	Control, Females	CUS, Females	Two-Way ANOVA, *p*-Level			*t*-Test, *p*-Level
	*n* = 14	*n* = 12	*n* = 13	*n* = 13	sex	group	Sex * group	
Thymus, mg (per 1 g of BW)	14.92 ± 4.11 (0.92)	16.83 ± 3.23 (0.99)	15.42 ± 3.11 (1.06)	17.88 ± 4.92 (1.17)	n.s. m < f	# S > C	n.s.	Sf > Cf n.s. Sm > Cm n.s. Sm < Sf n.s. Cm < Cf n.s.
Adrenal glands, mg (per 1 g of BW)	5.19 ± 1.26 (0.32)	6.36 ± 2.01 (0.37)	6.56 ± 1.25 (0.45)	7.25 ± 2.20 (0.47)	* m < f	# S > C	n.s.	Sf > Cf n.s. Sm > Cm # Sm < Sf n.s. Cm < Cf ** (d = −1.09)
BW, g	16.30 ± 2.20	16.97 ± 1.71	14.56 ± 2.08	15.34 ± 1.58	** m > f	n.s. S > C	n.s.	Sf > Cf n.s. Sm > Cm n.s. Sm > Sf * (d = 0.993) Cm > Cf * (d = 0.813)

Significance levels: ** *p* < 0.01, * *p* < 0.05, # *p* < 0.1, and n.s.—*p* > 0.1; m, males; f, females; S, CUS; C, control; BW, body weight at sacrifice; d = Cohen’s d for the significant effects. Full ANOVA and *t*-test results are available in Table S3 of the Supplementary Materials. Data are expressed as the mean ± SD.

## Data Availability

The original contributions presented in this study are included in the Supplementary Materials. Further inquiries can be directed to the corresponding author.

## Supplementary Materials

The following supporting information can be downloaded at https://www.mdpi.com/article/10.3390/biology15010054/s1: Table S1: Body weights of mice during the experiment, 2-way ANOVA results; Table S2: Body weight gain during the experiment, 2-way ANOVA and *t*-test results; Table S3: Post-mortem analysis of organ and body weights, 2-way ANOVA and *t*-test results; Table S4: Behavioral tests, Mann–Whitney test results and effect sizes; Table S5: Corticosterone levels after CUS exposure, Mann–Whitney test results and effect sizes; Table S6: Chronic unpredictable stress procedures; Figure S1: Spearman correlation matrix depicting pairwise associations between measured parameters; Table S7: Complete listing of Spearman correlation analyses presented in long format; Table S8: The results of MANOVA; Table S9: The ARRIVE guidelines 2.0: author checklist; row_data.xlsx.

## Abbreviations

The following abbreviations are used in this manuscript:

*M. m.*	*Mus musculus*
CUS	Chronic unpredictable stress
OFT	Open field test
TST	Tail suspension test
FST	Forced swim test
HPA	Hypothalamic–pituitary–adrenal
ELISA	Enzyme-linked immunosorbent assay
SD	Standard deviation
ANOVA	Analysis of variance
