# Supplementary material for: Sex Differences in Reaction to Chronic Unpredictable Stress in the House Mouse (Mus musculus musculus) of Wild Origin
Source: Biology (Basel). 2025 Dec 28;15(1):54. doi: 10.3390/biology15010054 (PMC12784703; doi:10.3390/biology15010054)
Supplement: Supplementary file 1 [file biology-15-00054-s001.zip › biology_supplementary_materials_rev2.pdf]

Table S1: Body weights of mice during the experiment, 2-way ANOVA results

ANOVA Table (type II tests) - body weight on Day 1, g

Effect	DFn	DFd	F	p	p<.05	ges
sex	1	48	8.042	0.007	*	0.143
group	1	48	5.793	0.020	*	0.108
sex:group	1	48	0.904	0.347		0.018

ANOVA Table (type II tests) - body weight on Day 15, g

Effect	DFn	DFd	F	p	p<.05	ges
sex	1	48	16.200	0.000201	*	0.252
group	1	48	0.389	0.536000		0.008
sex:group	1	48	0.265	0.609000		0.006

ANOVA Table (type II tests) - body weight on Day 29, g

Effect	DFn	DFd	F	p	p<.05	ges
sex	1	48	10.188	0.002	*	0.175000
group	1	48	0.010	0.922		0.000203
sex:group	1	48	0.011	0.917		0.000227

ANOVA Table (type II tests)- body weight on Day 36, g

Effect	DFn	DFd	F	p	p<.05	ges
sex	1	48	17.508	0.000121	*	0.267
group	1	48	0.971	0.329000		0.020
sex:group	1	48	0.104	0.749000		0.002

ges, generalized eta squared effect-size

Table S2: Body weight gain during the experiment, 2-way ANOVA & t-test results

ANOVA Table (type II tests) - body weight gain on Days 1-15, g

Effect	DFn	DFd	F	p	p<.05	ges
sex	1	48	7.439	9.00e-03	*	0.134
group	1	48	45.357	1.88e-08	*	0.486
sex:group	1	48	0.910	3.45e-01		0.019

Pairwise t-tests - body weight gain on Days 1-15, g

group1	group2	n1	n2	statistic	df	p	p.adj	p.adj.signif
Ctrl_f	Stress_f	13	13	7.11	21.9	4.02e-7	1.61e-6	****
Ctrl_m	Stress_m	14	12	3.57	21.3	2e-3	5e-3	**
Stress_f	Stress_m	13	12	-3.48	20.7	2e-3	5e-3	**
Ctrl_f	Ctrl_m	13	14	-1.11	21.8	2.79e-1	2.79e-1	ns

Cohen's d effect sizes - body weight gain on Days 1-15, g

group1	group2	effsize	n1	n2	magnitude
Ctrl_f	Stress_f	2.79	13	13	large
Ctrl_m	Stress_m	1.38	14	12	large
Stress_f	Stress_m	-1.40	13	12	large
Ctrl_f	Ctrl_m	-0.424	13	14	small

ANOVA Table (type II tests) - body weight gain on Days 15-29, g

Effect	DFn	DFd	F	p	p<.05	ges
sex	1	48	3.204	0.080		0.063
group	1	48	2.592	0.114		0.051

sex:group	1	48	1.568	0.217		0.032

Pairwise t-tests - body weight gain on Days 15-29

group1	group2	n1	n2	statistic	df	p	p.adj	p.adj.signif
Ctrl_f	Stress_f	13	13	-0.252	19.4	0.804	1	ns
Ctrl_m	Stress_m	14	12	-2.11	22.9	0.046	0.182	ns
Stress_f	Stress_m	13	12	0.451	22.5	0.656	1	ns
Ctrl_f	Ctrl_m	13	14	1.81	24.4	0.082	0.246	ns

Cohen's d effect sizes - body weight gain on Days 15-29, g

group1	group2	effsize	n1	n2	magnitude
Ctrl_f	Stress_f	-0.0989	13	13	negligible
Ctrl_m	Stress_m	-0.820	14	12	large
Stress_f	Stress_m	0.181	13	12	negligible
Ctrl_f	Ctrl_m	0.700	13	14	moderate

ANOVA Table (type II tests) - body weight gain on Days 29-36, g

Effect	DFn	DFd	F	p	p<.05	ges
sex	1	48	4.541	0.038	*	0.086
group	1	48	8.466	0.005	*	0.150
sex:group	1	48	0.318	0.575		0.007

Pairwise t-tests - body weight gain on Days 29-36, g

group1	group2	n1	n2	statistic	df	p	p.adj	p.adj.signif
Ctrl_f	Stress_f	13	13	-2.51	24.0	0.019	0.077	ns
Ctrl_m	Stress_m	14	12	-1.54	15.4	0.143	0.286	ns
Stress_f	Stress_m	13	12	-0.910	20.2	0.374	0.374	ns
Ctrl_f	Ctrl_m	13	14	-2.31	20.7	0.031	0.094	ns

Cohen's d effect sizes - body weight gain on Days 29-36, g

group1	group2	effsize	n1	n2	magnitude
Ctrl_f	Stress_f	-0.985	13	13	large
Ctrl_m	Stress_m	-0.622	14	12	moderate
Stress_f	Stress_m	-0.366	13	12	small
Ctrl_f	Ctrl_m	-0.896	13	14	large

ANOVA Table (type II tests) - body weight gain on Days 1-36, g

Effect	DFn	DFd	F	p	p<.05	ges
sex	1	48	7.495	0.009	*	0.135
group	1	48	6.722	0.013	*	0.123
sex:group	1	48	1.328	0.255		0.027

Pairwise t-tests - body weight gain on Days 1-36, g

group1	group2	n1	n2	statistic	df	p	p.adj	p.adj.signif
Ctrl_f	Stress_f	13	13	3.36	24.0	0.003	0.01	*
Ctrl_m	Stress_m	14	12	0.891	22.8	0.382	0.598	ns
Stress_f	Stress_m	13	12	-3.21	21.7	0.004	0.012	*
Ctrl_f	Ctrl_m	13	14	-1.06	21.4	0.299	0.598	ns

Cohen's d effect sizes - body weight gain on Days 1-36, g

group1	group2	effsize	n1	n2	magnitude
Ctrl_f	Stress_f	1.32	13	13	large
Ctrl_m	Stress_m	0.345	14	12	small
Stress_f	Stress_m	-1.29	13	12	large
Ctrl_f	Ctrl_m	-0.406	13	14	small

ges, generalized eta squared effect-size; p.adj, p values adjusted for multiple comparisons using Holm method

Table S3: *Post mortem* analysis of organ and body weights, 2-way ANOVA & t-test results

ANOVA Table (type II tests) - thymus weight, mg

Effect	DFn	DFd	F	p	p<.05	ges
sex	1	48	0.488	0.488		0.010
group	1	48	4.002	0.051		0.077
sex:group	1	48	0.062	0.805		0.001

Pairwise t-tests - thymus weight, mg

group1	group2	n1	n2	statistic	df	p	p.adj	p.adj.signif
Ctrl_f	Stress_f	13	13	-1.52	20.3	0.144	0.576	ns
Ctrl_m	Stress_m	14	12	-1.33	23.8	0.197	0.591	ns
Stress_f	Stress_m	13	12	0.631	20.8	0.535	1	ns
Ctrl_f	Ctrl_m	13	14	0.359	24.0	0.723	1	ns

Cohen's d effect sizes - thymus weight, mg

group1	group2	effsize	n1	n2	magnitude
Ctrl_f	Stress_f	-0.596	13	13	moderate
Ctrl_m	Stress_m	-0.517	14	12	moderate
Stress_f	Stress_m	0.251	13	12	small
Ctrl_f	Ctrl_m	0.138	13	14	negligible

ANOVA Table (type II tests) - adrenal weight, mg

Effect	DFn	DFd	F	p	p<.05	ges
sex	1	48	5.680	0.021	*	0.106
group	1	48	3.745	0.059		0.072
sex:group	1	48	0.253	0.617		0.005

Pairwise t-tests - adrenal weight, mg

group1	group2	n1	n2	statistic	df	p	p.adj	p.adj.signif
Ctrl_f	Stress_f	13	13	-0.976	19.0	0.341	0.606	ns
Ctrl_m	Stress_m	14	12	-1.74	18.0	0.1	0.299	ns
Stress_f	Stress_m	13	12	1.05	23.0	0.303	0.606	ns
Ctrl_f	Ctrl_m	13	14	2.83	24.9	0.009	0.036	*

Cohen's d effect sizes - adrenal weight, mg

group1	group2	effsize	n1	n2	magnitude
Ctrl_f	Stress_f	-0.383	13	13	small
Ctrl_m	Stress_m	-0.694	14	12	moderate
Stress_f	Stress_m	0.421	13	12	small
Ctrl_f	Ctrl_m	1.09	13	14	large

ANOVA Table (type II tests) - body weight at sacrifice, g

Effect	DFn	DFd	F	p	p<.05	ges
sex	1	48	10.058	0.003	*	0.173000
group	1	48	1.835	0.182		0.037000
sex:group	1	48	0.010	0.920		0.000211

Pairwise t-tests - body weight at sacrifice, g

group1	group2	n1	n2	statistic	df	p	p.adj	p.adj.signif
Ctrl_f	Stress_f	13	13	-1.07	22.3	0.295	0.59	ns
Ctrl_m	Stress_m	14	12	-0.871	23.8	0.393	0.59	ns
Stress_f	Stress_m	13	12	-2.48	22.4	0.021	0.085	ns
Ctrl_f	Ctrl_m	13	14	-2.11	25.0	0.045	0.134	ns

Cohen's d effect sizes - body weight at sacrifice, g

group1	group2	effsize	n1	n2	magnitude
Ctrl_f	Stress_f	-0.420	13	13	small
Ctrl_m	Stress_m	-0.339	14	12	small
Stress_f	Stress_m	-0.993	13	12	large
Ctrl_f	Ctrl_m	-0.813	13	14	large

ges, generalized eta squared effect-size; p.adj, p values adjusted for multiple comparisons using Holm method

Table S4: Behavioral tests, Mann-Whitney test results & effect sizes

Wilcoxon tests - entries to center in OFT, n

group1	group2	n1	n2	statistic	p	p.adj	p.adj.signif
Ctrl_f	Stress_f	13	13	84	1	1	ns
Ctrl_m	Stress_m	14	12	74.5	0.643	1	ns
Stress_f	Stress_m	13	12	81.5	0.87	1	ns
Ctrl_f	Ctrl_m	13	14	99.5	0.698	1	ns
f	m	26	26	368	0.589		
Ctrl	Stress	27	25	314	0.68		

Wilcoxon effect sizes - entries to center in OFT, n

group1	group2	effsize	n1	n2	magnitude
Ctrl_f	Stress_f	0.00503	13	13	small
Ctrl_m	Stress_m	0.0959	14	12	small
Stress_f	Stress_m	0.0381	13	12	small
Ctrl_f	Ctrl_m	0.0794	13	14	small
f	m	0.0762	26	26	small
Ctrl	Stress	0.0584	27	25	small

Wilcoxon tests - defecation boluses in OFT, n

group1	group2	n1	n2	statistic	p	p.adj	p.adj.signif
Ctrl_f	Stress_f	13	13	76	0.681	0.681	ns
Ctrl_m	Stress_m	14	12	59.5	0.215	0.474	ns
Stress_f	Stress_m	13	12	45.5	0.081	0.324	ns
Ctrl_f	Ctrl_m	13	14	61.5	0.158	0.474	ns
f	m	26	26	213	0.0224		
Ctrl	Stress	27	25	266	0.196		

Wilcoxon effect sizes - defecation boluses in OFT, n

group1	group2	effsize	n1	n2	magnitude
Ctrl_f	Stress_f	0.0858	13	13	small
Ctrl_m	Stress_m	0.248	14	12	small
Stress_f	Stress_m	0.354	13	12	moderate
Ctrl_f	Ctrl_m	0.277	13	14	small
f	m	0.318	26	26	moderate
Ctrl	Stress	0.181	27	25	small

Wilcoxon tests - grooming in OFT, sec

group1	group2	n1	n2	statistic	p	p.adj	p.adj.signif
Ctrl_f	Stress_f	13	13	43	0.035	0.142	ns
Ctrl_m	Stress_m	14	12	91	0.742	1	ns
Stress_f	Stress_m	13	12	85.5	0.703	1	ns
Ctrl_f	Ctrl_m	13	14	51	0.055	0.166	ns
f	m	26	26	268	0.207		
Ctrl	Stress	27	25	264	0.181		

Wilcoxon effect sizes - grooming in OFT, sec

group1	group2	effsize	n1	n2	magnitude
Ctrl_f	Stress_f	0.417	13	13	moderate
Ctrl_m	Stress_m	0.0706	14	12	small
Stress_f	Stress_m	0.0816	13	12	small
Ctrl_f	Ctrl_m	0.374	13	14	moderate
f	m	0.176	26	26	small
Ctrl	Stress	0.187	27	25	small

Wilcoxon tests - rearing in OFT, n

group1	group2	n1	n2	statistic	p	p.adj	p.adj.signif
Ctrl_f	Stress_f	13	13	79	0.798	1	ns
Ctrl_m	Stress_m	14	12	91	0.738	1	ns
Stress_f	Stress_m	13	12	113	0.06	0.242	ns
Ctrl_f	Ctrl_m	13	14	105	0.519	1	ns
f	m	26	26	425	0.113		
Ctrl	Stress	27	25	329	0.884		

Wilcoxon effect sizes - rearing in OFT, n

group1	group2	effsize	n1	n2	magnitude
Ctrl_f	Stress_f	0.0553	13	13	small
Ctrl_m	Stress_m	0.0706	14	12	small
Stress_f	Stress_m	0.381	13	12	moderate
Ctrl_f	Ctrl_m	0.131	13	14	small
f	m	0.221	26	26	small
Ctrl	Stress	0.0216	27	25	small

Wilcoxon tests - hole pokes in OFT, n

group1	group2	n1	n2	statistic	p	p.adj	p.adj.signif
Ctrl_f	Stress_f	13	13	76.5	0.699	1	ns
Ctrl_m	Stress_m	14	12	109	0.205	0.82	ns
Stress_f	Stress_m	13	12	76	0.934	1	ns
Ctrl_f	Ctrl_m	13	14	68.5	0.284	0.852	ns
f	m	26	26	290	0.378		
Ctrl	Stress	27	25	362	0.652		

Wilcoxon effect sizes - hole pokes in OFT, n

group1	group2	effsize	n1	n2	magnitude
Ctrl_f	Stress_f	0.0808	13	13	small
Ctrl_m	Stress_m	0.254	14	12	small
Stress_f	Stress_m	0.0219	13	12	small
Ctrl_f	Ctrl_m	0.211	13	14	small
f	m	0.124	26	26	small
Ctrl	Stress	0.0638	27	25	small

Wilcoxon tests – immobility time in OFT, n

group1	group2	n1	n2	statistic	p	p.adj	p.adj.signif
Ctrl_f	Stress_f	13	13	92.5	0.7	1	ns
Ctrl_m	Stress_m	14	12	71	0.52	1	ns
Stress_f	Stress_m	13	12	77.5	1	1	ns
Ctrl_f	Ctrl_m	13	14	110	0.382	1	ns
f	m	26	26	373	0.528		
Ctrl	Stress	27	25	330	0.891		

Wilcoxon effect sizes – immobility time in OFT, n

group1	group2	effsize	n1	n2	magnitude
Ctrl_f	Stress_f	0.0805	13	13	small

Ctrl_m	Stress_m	0.131	14	12	small
Stress_f	Stress_m	0.00544	13	12	small
Ctrl_f	Ctrl_m	0.173	13	14	small
f	m	0.0889	26	26	small
Ctrl	Stress	0.0203	27	25	small

Wilcoxon tests - latency to the 1st immobility in TST, sec

group1	group2	n1	n2	statistic	p	p.adj	p.adj.signif
Ctrl_f	Stress_f	12	13	78	1	1	ns
Ctrl_m	Stress_m	14	12	66.5	0.357	1	ns
Stress_f	Stress_m	13	12	55.5	0.204	0.816	ns
Ctrl_f	Ctrl_m	12	14	78.5	0.779	1	ns
f	m	25	26	274	0.307		
Ctrl	Stress	26	25	298	0.585		

Wilcoxon effect sizes - latency to the 1st immobility in TST, sec

group1	group2	effsize	n1	n2	magnitude
Ctrl_f	Stress_f	0	12	13	small
Ctrl_m	Stress_m	0.186	14	12	small
Stress_f	Stress_m	0.260	13	12	small
Ctrl_f	Ctrl_m	0.0604	12	14	small
f	m	0.145	25	26	small
Ctrl	Stress	0.0779	26	25	small

Wilcoxon tests - immobility time in TST, sec

group1	group2	n1	n2	statistic	p	p.adj	p.adj.signif
Ctrl_f	Stress_f	9	10	34	0.4	0.8	ns
Ctrl_m	Stress_m	13	12	116	0.044	0.132	ns
Stress_f	Stress_m	10	12	93.5	0.029	0.118	ns
Ctrl_f	Ctrl_m	9	13	45.5	0.404	0.8	ns
f	m	19	25	270	0.455		
Ctrl	Stress	22	22	275	0.445		

Wilcoxon effect sizes - immobility time in TST, sec

group1	group2	effsize	n1	n2	magnitude
Ctrl_f	Stress_f	0.206	9	10	small
Ctrl_m	Stress_m	0.408	13	12	moderate
Stress_f	Stress_m	0.471	10	12	moderate
Ctrl_f	Ctrl_m	0.185	9	13	small
f	m	0.114	19	25	small
Ctrl	Stress	0.117	22	22	small

Wilcoxon tests - immobility episodes in TST, n

group1	group2	n1	n2	statistic	p	p.adj	p.adj.signif
Ctrl_f	Stress_f	9	10	18	0.03	0.12	ns
Ctrl_m	Stress_m	13	12	100	0.23	0.282	ns
Stress_f	Stress_m	10	12	87.5	0.075	0.224	ns
Ctrl_f	Ctrl_m	9	13	36	0.141	0.282	ns
f	m	19	25	264	0.529		
Ctrl	Stress	22	22	214	0.51		

Wilcoxon effect sizes - immobility episodes in TST, n

group1	group2	effsize	n1	n2	magnitude
Ctrl_f	Stress_f	0.508	9	10	large
Ctrl_m	Stress_m	0.245	13	12	small
Stress_f	Stress_m	0.387	10	12	moderate

Ctrl_f	Ctrl_m	0.321	9	13	moderate
f	m	0.0966	19	25	small
Ctrl	Stress	0.101	22	22	small

Ctrl_f, control females; Stress_f, stressed females; Ctrl_m, control males; Stress_m, stressed males; p.adj, p values adjusted for multiple comparisons using Holm method

Table S5: Corticosterone levels after CUS exposure, Mann-Whitney test results & effect sizes

Wilcoxon tests - hair corticosterone

group1	group2	n1	n2	statistic	p	p.adj	p.adj.signif
Ctrl_f	Stress_f	13	13	90	0.801	0.94	ns
Ctrl_m	Stress_m	14	12	43	0.036	0.107	ns
Stress_f	Stress_m	13	12	92	0.47	0.94	ns
Ctrl_f	Ctrl_m	13	14	138	0.022	0.089	ns
f	m	26	26	450	0.0406		
Ctrl	Stress	27	25	275	0.259		

Wilcoxon effect sizes – hair corticosterone

group1	group2	effsize	n1	n2	magnitude
Ctrl_f	Stress_f	0.0553	13	13	small
Ctrl_m	Stress_m	0.414	14	12	moderate
Stress_f	Stress_m	0.152	13	12	small
Ctrl_f	Ctrl_m	0.439	13	14	moderate
f	m	0.284	26	26	small
Ctrl	Stress	0.159	27	25	small

Wilcoxon tests - plasma corticosterone

group1	group2	n1	n2	statistic	p	p.adj	p.adj.signif
Ctrl_f	Stress_f	11	12	71	0.786	1	ns
Ctrl_m	Stress_m	11	11	34	0.088	0.264	ns
Stress_f	Stress_m	12	11	70	0.833	1	ns
Ctrl_f	Ctrl_m	11	11	94	0.028	0.112	ns
f	m	23	22	338	0.0544		
Ctrl	Stress	22	23	203	0.264		

Wilcoxon effect sizes – plasma corticosterone

group1	group2	effsize	n1	n2	magnitude
Ctrl_f	Stress_f	0.0642	11	12	small
Ctrl_m	Stress_m	0.371	11	11	moderate
Stress_f	Stress_m	0.0513	12	11	small
Ctrl_f	Ctrl_m	0.469	11	11	moderate
f	m	0.288	23	22	small
Ctrl	Stress	0.169	22	23	small

Ctrl_f, control females; Stress_f, stressed females; Ctrl_m, control males; Stress_m, stressed males;; p.adj, p values adjusted for multiple comparisons using Holm method

Table S6: Chronic unpredictable stress procedures.

	Day 1	Day 2	Day 3	Day 4	Day 5	Day 6	Day 7
**Week 1**	FD (night)	CAGE TILT (evening)	CAT ODOR (day)	ODOR RATS (day)	RESTRAINT (morning)	LIGHT NIGHT (night)	RESTRAINT (day)
**Week 2**	CAT ODOR (day)	WD (night)	RESTRAINT (day)	ODOR RATS (day)	COLD (day)	WET BEDDING (morning – day)	CAGE TILT (morning)
**Week 3**	FD (night)	CAGE TILT (evening)	RESTRAINT (morning)	LIGHT NIGHT (night)	CAT ODOR (day)	RESTRAINT (morning)	ODOR RATS (day)
**Week 4**	WD (night)	COLD (evening)	CAT ODOR (morning)	CAGE TILT (day)	RESTRAINT (morning)	WET BEDDING (morning – day)	HOT AIR (morning), ODOR RATS (day)
**Week 5**	FD (night)	CAGE TILT (evening)	ODOR RATS (day)	RESTRAINT (day)	LIGHT NIGHT (night)	COLD (evening)	CAT ODOR (day); On day 36 mice were weighed.

The following stressors were included in the protocol: restraint in 50 mL conical tubes for one hour (RESTRAINT, 7 times), wet bedding for twelve hours (WET BEDDING, 2 times), cage tilted at 45 degrees for one hour (CAGE TILT, 5 times), lights on overnight (LIGHT NIGHT, 3 times), cold chamber at 4° C for one hour (COLD, 3 times), water or food deprivation for sixteen hours (WD, 2 times; FD, 3 times), hot air stream for fifteen minutes (HOT AIR, 1 time), exposure to cat urine odor 100 µl (CAT ODOR, 5 times) or exposure to rats (Rattus norvegicus) for one hour (ODOR RATS, 5 times). Fresh cat urine was collected from an adult unneutered female cat and stored at −40 °C until use. For exposure to rats, cages with mice were moved to the room where the laboratory rat colony was maintained. One or two stimulation patterns were applied daily. Stress exposures started at different times between 8 am and 9 pm. The chronic stress protocol schedule was designed to ensure unpredictability and manage cumulative severity per week. Each mouse was exposed to specific stressors on a weekly basis: one session of cat odor, one of rat odor, one of cage tilt, one of food or water deprivation. Furthermore, each mouse underwent either two restraint sessions or one restraint and one cold stress session per week. Food or water deprivation was completed at least five days prior to any weight assessment. Finally, the schedule was constrained to the operating hours of the animal facility, so that most stressors were applied during the light phase.

Each mouse underwent a total of 36 stress exposure sessions, administered according to the schedule outlined above. Animals were distributed into four balanced cohorts; all mice within a given cohort were subjected to the stress protocol on the same designated day according to the experimental schedule. Behavioral tests were conducted following a 1-4 day rest period after completion of the CUS procedure; a period of rest (1-4 days) was implemented to assess the accumulated long-term effects of the chronic stress protocol, rather than the acute response to the most recent stressor.

Animals were daily monitored for normal appearance: posture, fur quality, eyes. Exclusion criteria of moderate to severe appearance abnormalities, and/or body weight loss of >15% from baseline were not met in our study.

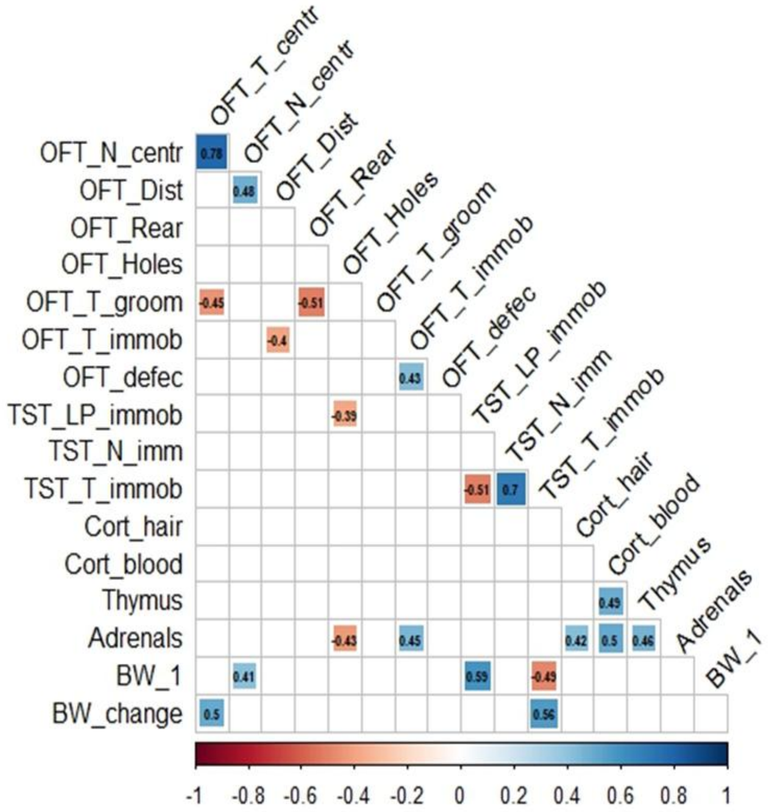

(**a**) males

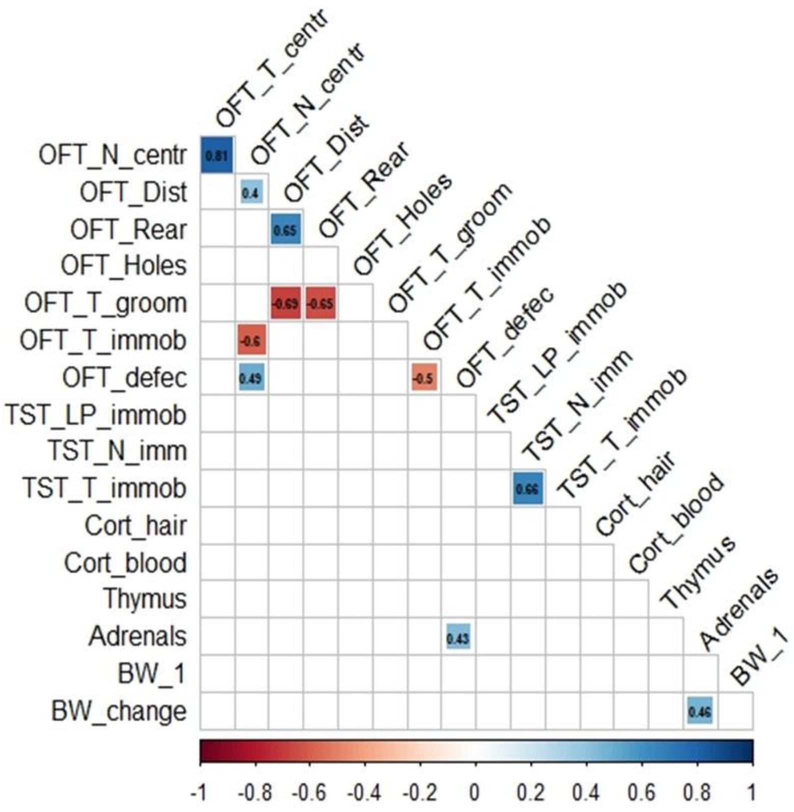

(**b**) females

Figure S1. Spearman correlation matrix depicting pairwise associations between measured parameters. Analyses were performed separately in males (a) and females (b). Correlation coefficients are color-coded according to the scale. Only statistically significant associations ($p < 0.05$) are displayed; non-significant correlations appear as empty cells.

Table S7 Complete listing of Spearman correlation analyses presented in long format.

Variable 1	Variable 2	Males			Females		
		Rs	p-value	N	Rs	p-value	N
OFT_T_centr	OFT_T_centr	1	0	26	1	0	26
OFT_N_centr	OFT_T_centr	0.78	3.16E-06	26	0.81	5.58E-07	26
OFT_Dist	OFT_T_centr	0.25	0.217	26	0.11	0.582	26
OFT_Rear	OFT_T_centr	0.17	0.42	26	0.085	0.679	26
OFT_Holes	OFT_T_centr	0.28	0.171	26	0.16	0.423	26
OFT_T_groom	OFT_T_centr	-0.45	0.0206	26	-0.07	0.732	26
OFT_T_immob	OFT_T_centr	-0.018	0.932	26	-0.36	0.0737	26
OFT_defec	OFT_T_centr	0.28	0.168	26	0.2	0.317	26
TST_LP_immob	OFT_T_centr	0.046	0.822	26	-0.22	0.288	25
TST_N_imm	OFT_T_centr	0.17	0.411	25	0.27	0.258	19
TST_T_immob	OFT_T_centr	0.26	0.204	25	0.29	0.222	19
Cort_hair	OFT_T_centr	0.27	0.191	26	0.034	0.871	26
Cort_blood	OFT_T_centr	0.21	0.34	22	0.19	0.372	23
Thymus	OFT_T_centr	0.28	0.16	26	0.3	0.134	26
Adrenals	OFT_T_centr	-0.2	0.322	26	0.16	0.439	26
BW_1	OFT_T_centr	0.22	0.273	26	-0.12	0.575	26
BW_change	OFT_T_centr	0.5	0.00851	26	0.071	0.73	26
OFT_T_centr	OFT_N_centr	0.78	3.16E-06	26	0.81	5.58E-07	26
OFT_N_centr	OFT_N_centr	1	2.32E-189	26	1	0	26
OFT_Dist	OFT_N_centr	0.48	0.0137	26	0.4	0.0405	26
OFT_Rear	OFT_N_centr	0.28	0.165	26	0.33	0.0971	26
OFT_Holes	OFT_N_centr	0.1	0.618	26	0.23	0.261	26
OFT_T_groom	OFT_N_centr	-0.37	0.0634	26	-0.3	0.131	26
OFT_T_immob	OFT_N_centr	-0.03	0.884	26	-0.6	0.0013	26
OFT_defec	OFT_N_centr	0.29	0.151	26	0.49	0.0117	26
TST_LP_immob	OFT_N_centr	0.21	0.314	26	-0.025	0.906	25
TST_N_imm	OFT_N_centr	0.29	0.16	25	0.38	0.106	19
TST_T_immob	OFT_N_centr	0.16	0.446	25	0.14	0.559	19
Cort_hair	OFT_N_centr	0.28	0.162	26	0.14	0.494	26
Cort_blood	OFT_N_centr	0.28	0.211	22	0.18	0.412	23
Thymus	OFT_N_centr	0.22	0.279	26	0.19	0.351	26
Adrenals	OFT_N_centr	-0.046	0.824	26	0.37	0.0667	26
BW_1	OFT_N_centr	0.41	0.0387	26	0.061	0.766	26
BW_change	OFT_N_centr	0.14	0.494	26	0.22	0.286	26
OFT_T_centr	OFT_Dist	0.25	0.217	26	0.11	0.582	26
OFT_N_centr	OFT_Dist	0.48	0.0137	26	0.4	0.0405	26
OFT_Dist	OFT_Dist	1	8.88E-08	26	1	8.88E-08	26
OFT_Rear	OFT_Dist	0.37	0.0636	26	0.65	0.000286	26
OFT_Holes	OFT_Dist	-0.00069	0.997	26	-0.054	0.792	26
OFT_T_groom	OFT_Dist	-0.31	0.127	26	-0.69	9.49E-05	26
OFT_T_immob	OFT_Dist	-0.4	0.042	26	-0.34	0.0851	26
OFT_defec	OFT_Dist	0.2	0.331	26	0.2	0.326	26
TST_LP_immob	OFT_Dist	0.23	0.268	26	0.039	0.853	25
TST_N_imm	OFT_Dist	-0.14	0.502	25	0.17	0.494	19
TST_T_immob	OFT_Dist	-0.11	0.588	25	0.21	0.398	19

Cort_hair	OFT_Dist	0.11	0.604	26	0.28	0.163	26
Cort_blood	OFT_Dist	0.14	0.538	22	-0.028	0.901	23
Thymus	OFT_Dist	-0.26	0.206	26	0.058	0.779	26
Adrenals	OFT_Dist	-0.28	0.158	26	0.17	0.402	26
BW_1	OFT_Dist	0.11	0.577	26	-0.074	0.718	26
BW_change	OFT_Dist	-0.21	0.298	26	0.057	0.783	26
OFT_T_centr	OFT_Rear	0.17	0.42	26	0.085	0.679	26
OFT_N_centr	OFT_Rear	0.28	0.165	26	0.33	0.0971	26
OFT_Dist	OFT_Rear	0.37	0.0636	26	0.65	0.000286	26
OFT_Rear	OFT_Rear	1	0	26	1	0	26
OFT_Holes	OFT_Rear	0.17	0.414	26	-0.21	0.306	26
OFT_T_groom	OFT_Rear	-0.51	0.00785	26	-0.65	0.000364	26
OFT_T_immob	OFT_Rear	-0.36	0.0742	26	-0.35	0.084	26
OFT_defec	OFT_Rear	-0.095	0.643	26	0.27	0.186	26
TST_LP_immob	OFT_Rear	0.34	0.0908	26	-0.16	0.459	25
TST_N_imm	OFT_Rear	0.12	0.571	25	0.15	0.536	19
TST_T_immob	OFT_Rear	-0.15	0.473	25	-0.025	0.922	19
Cort_hair	OFT_Rear	-0.068	0.741	26	0.17	0.408	26
Cort_blood	OFT_Rear	0.077	0.732	22	-0.00049	0.998	23
Thymus	OFT_Rear	-0.034	0.87	26	-0.09	0.662	26
Adrenals	OFT_Rear	-0.3	0.132	26	0.16	0.421	26
BW_1	OFT_Rear	0.38	0.0527	26	-0.03	0.883	26
BW_change	OFT_Rear	-0.28	0.167	26	-0.055	0.791	26
OFT_T_centr	OFT_Holes	0.28	0.171	26	0.16	0.423	26
OFT_N_centr	OFT_Holes	0.1	0.618	26	0.23	0.261	26
OFT_Dist	OFT_Holes	-0.00069	0.997	26	-0.054	0.792	26
OFT_Rear	OFT_Holes	0.17	0.414	26	-0.21	0.306	26
OFT_Holes	OFT_Holes	1	0	26	1	0	26
OFT_T_groom	OFT_Holes	0.039	0.851	26	-0.025	0.904	26
OFT_T_immob	OFT_Holes	-0.3	0.14	26	-0.37	0.0613	26
OFT_defec	OFT_Holes	-0.2	0.328	26	-0.038	0.855	26
TST_LP_immob	OFT_Holes	-0.39	0.0462	26	0.16	0.445	25
TST_N_imm	OFT_Holes	0.29	0.154	25	-0.14	0.569	19
TST_T_immob	OFT_Holes	0.31	0.138	25	-0.26	0.29	19
Cort_hair	OFT_Holes	-0.0028	0.989	26	0.044	0.83	26
Cort_blood	OFT_Holes	-0.32	0.146	22	-0.13	0.564	23
Thymus	OFT_Holes	0.13	0.513	26	-0.096	0.64	26
Adrenals	OFT_Holes	-0.43	0.0283	26	0.018	0.931	26
BW_1	OFT_Holes	-0.17	0.41	26	0.19	0.35	26
BW_change	OFT_Holes	0.13	0.543	26	0.062	0.763	26
OFT_T_centr	OFT_T_groom	-0.45	0.0206	26	-0.07	0.732	26
OFT_N_centr	OFT_T_groom	-0.37	0.0634	26	-0.3	0.131	26
OFT_Dist	OFT_T_groom	-0.31	0.127	26	-0.69	9.49E-05	26
OFT_Rear	OFT_T_groom	-0.51	0.00785	26	-0.65	0.000364	26
OFT_Holes	OFT_T_groom	0.039	0.851	26	-0.025	0.904	26
OFT_T_groom	OFT_T_groom	1	8.88E-08	26	1	0	26
OFT_T_immob	OFT_T_groom	0.14	0.51	26	0.32	0.111	26
OFT_defec	OFT_T_groom	-0.24	0.237	26	-0.15	0.468	26

TST_LP_immob	OFT_T_groom	-0.19	0.356	26	-0.07	0.74	25
TST_N_imm	OFT_T_groom	0.011	0.959	25	0.33	0.172	19
TST_T_immob	OFT_T_groom	-0.074	0.726	25	0.23	0.34	19
Cort_hair	OFT_T_groom	-0.22	0.281	26	-0.35	0.0824	26
Cort_blood	OFT_T_groom	-0.13	0.558	22	0.12	0.595	23
Thymus	OFT_T_groom	-0.017	0.933	26	0.3	0.142	26
Adrenals	OFT_T_groom	0.21	0.297	26	-0.3	0.143	26
BW_1	OFT_T_groom	-0.22	0.275	26	0.3	0.135	26
BW_change	OFT_T_groom	-0.04	0.846	26	-0.34	0.0876	26
OFT_T_centr	OFT_T_immob	-0.018	0.932	26	-0.36	0.0737	26
OFT_N_centr	OFT_T_immob	-0.03	0.884	26	-0.6	0.0013	26
OFT_Dist	OFT_T_immob	-0.4	0.042	26	-0.34	0.0851	26
OFT_Rear	OFT_T_immob	-0.36	0.0742	26	-0.35	0.084	26
OFT_Holes	OFT_T_immob	-0.3	0.14	26	-0.37	0.0613	26
OFT_T_groom	OFT_T_immob	0.14	0.51	26	0.32	0.111	26
OFT_T_immob	OFT_T_immob	1	0	26	1	2.32E-189	26
OFT_defec	OFT_T_immob	0.43	0.0273	26	-0.5	0.00941	26
TST_LP_immob	OFT_T_immob	0.012	0.955	26	0.099	0.638	25
TST_N_imm	OFT_T_immob	-0.28	0.172	25	-0.1	0.68	19
TST_T_immob	OFT_T_immob	-0.37	0.0721	25	0.1	0.676	19
Cort_hair	OFT_T_immob	0.21	0.298	26	-0.24	0.232	26
Cort_blood	OFT_T_immob	0.16	0.485	22	0.043	0.844	23
Thymus	OFT_T_immob	0.23	0.261	26	0.097	0.639	26
Adrenals	OFT_T_immob	0.45	0.0223	26	-0.14	0.505	26
BW_1	OFT_T_immob	0.19	0.361	26	-0.034	0.871	26
BW_change	OFT_T_immob	-0.098	0.634	26	-0.1	0.62	26
OFT_T_centr	OFT_defec	0.28	0.168	26	0.2	0.317	26
OFT_N_centr	OFT_defec	0.29	0.151	26	0.49	0.0117	26
OFT_Dist	OFT_defec	0.2	0.331	26	0.2	0.326	26
OFT_Rear	OFT_defec	-0.095	0.643	26	0.27	0.186	26
OFT_Holes	OFT_defec	-0.2	0.328	26	-0.038	0.855	26
OFT_T_groom	OFT_defec	-0.24	0.237	26	-0.15	0.468	26
OFT_T_immob	OFT_defec	0.43	0.0273	26	-0.5	0.00941	26
OFT_defec	OFT_defec	1	0	26	1	2.32E-189	26
TST_LP_immob	OFT_defec	0.097	0.636	26	0.036	0.865	25
TST_N_imm	OFT_defec	-0.2	0.347	25	0.31	0.199	19
TST_T_immob	OFT_defec	-0.15	0.477	25	0.082	0.739	19
Cort_hair	OFT_defec	0.24	0.233	26	-0.25	0.221	26
Cort_blood	OFT_defec	0.42	0.0545	22	-0.064	0.772	23
Thymus	OFT_defec	-0.044	0.832	26	0.0091	0.965	26
Adrenals	OFT_defec	0.16	0.435	26	0.43	0.0294	26
BW_1	OFT_defec	0.0048	0.981	26	0.16	0.449	26
BW_change	OFT_defec	0.12	0.573	26	0.36	0.0719	26
OFT_T_centr	TST_LP_immob	0.046	0.822	26	-0.22	0.288	25
OFT_N_centr	TST_LP_immob	0.21	0.314	26	-0.025	0.906	25
OFT_Dist	TST_LP_immob	0.23	0.268	26	0.039	0.853	25
OFT_Rear	TST_LP_immob	0.34	0.0908	26	-0.16	0.459	25
OFT_Holes	TST_LP_immob	-0.39	0.0462	26	0.16	0.445	25

OFT_T_groom	TST_LP_immob	-0.19	0.356	26	-0.07	0.74	25
OFT_T_immob	TST_LP_immob	0.012	0.955	26	0.099	0.638	25
OFT_defec	TST_LP_immob	0.097	0.636	26	0.036	0.865	25
TST_LP_immob	TST_LP_immob	1	0	26	1	1.59E-181	25
TST_N_imm	TST_LP_immob	-0.27	0.186	25	-0.13	0.608	19
TST_T_immob	TST_LP_immob	-0.51	0.00891	25	-0.35	0.142	19
Cort_hair	TST_LP_immob	0.1	0.622	26	0.042	0.842	25
Cort_blood	TST_LP_immob	0.38	0.0793	22	-0.13	0.569	22
Thymus	TST_LP_immob	0.14	0.503	26	-0.18	0.397	25
Adrenals	TST_LP_immob	0.24	0.243	26	0.22	0.286	25
BW_1	TST_LP_immob	0.59	0.00155	26	0.19	0.358	25
BW_change	TST_LP_immob	-0.23	0.26	26	0.072	0.732	25
OFT_T_centr	TST_N_imm	0.17	0.411	25	0.27	0.258	19
OFT_N_centr	TST_N_imm	0.29	0.16	25	0.38	0.106	19
OFT_Dist	TST_N_imm	-0.14	0.502	25	0.17	0.494	19
OFT_Rear	TST_N_imm	0.12	0.571	25	0.15	0.536	19
OFT_Holes	TST_N_imm	0.29	0.154	25	-0.14	0.569	19
OFT_T_groom	TST_N_imm	0.011	0.959	25	0.33	0.172	19
OFT_T_immob	TST_N_imm	-0.28	0.172	25	-0.1	0.68	19
OFT_defec	TST_N_imm	-0.2	0.347	25	0.31	0.199	19
TST_LP_immob	TST_N_imm	-0.27	0.186	25	-0.13	0.608	19
TST_N_imm	TST_N_imm	1	0	25	1	6.08E-132	19
TST_T_immob	TST_N_imm	0.7	9.16E-05	25	0.66	0.0021	19
Cort_hair	TST_N_imm	0.054	0.799	25	-0.21	0.393	19
Cort_blood	TST_N_imm	-0.26	0.247	21	0.078	0.773	16
Thymus	TST_N_imm	0.043	0.84	25	0.31	0.191	19
Adrenals	TST_N_imm	0.0039	0.985	25	0.17	0.49	19
BW_1	TST_N_imm	-0.053	0.8	25	0.072	0.769	19
BW_change	TST_N_imm	0.26	0.201	25	-0.027	0.912	19
OFT_T_centr	TST_T_immob	0.26	0.204	25	0.29	0.222	19
OFT_N_centr	TST_T_immob	0.16	0.446	25	0.14	0.559	19
OFT_Dist	TST_T_immob	-0.11	0.588	25	0.21	0.398	19
OFT_Rear	TST_T_immob	-0.15	0.473	25	-0.025	0.922	19
OFT_Holes	TST_T_immob	0.31	0.138	25	-0.26	0.29	19
OFT_T_groom	TST_T_immob	-0.074	0.726	25	0.23	0.34	19
OFT_T_immob	TST_T_immob	-0.37	0.0721	25	0.1	0.676	19
OFT_defec	TST_T_immob	-0.15	0.477	25	0.082	0.739	19
TST_LP_immob	TST_T_immob	-0.51	0.00891	25	-0.35	0.142	19
TST_N_imm	TST_T_immob	0.7	9.16E-05	25	0.66	0.0021	19
TST_T_immob	TST_T_immob	1	0	25	1	8.38E-06	19
Cort_hair	TST_T_immob	0.03	0.888	25	-0.21	0.398	19
Cort_blood	TST_T_immob	-0.26	0.261	21	0.23	0.391	16
Thymus	TST_T_immob	-0.14	0.497	25	0.1	0.678	19
Adrenals	TST_T_immob	-0.18	0.397	25	-0.11	0.659	19
BW_1	TST_T_immob	-0.49	0.0122	25	-0.28	0.25	19
BW_change	TST_T_immob	0.56	0.00353	25	0.12	0.614	19
OFT_T_centr	Cort_hair	0.27	0.191	26	0.034	0.871	26
OFT_N_centr	Cort_hair	0.28	0.162	26	0.14	0.494	26

OFT_Dist	Cort_hair	0.11	0.604	26	0.28	0.163	26
OFT_Rear	Cort_hair	-0.068	0.741	26	0.17	0.408	26
OFT_Holes	Cort_hair	-0.0028	0.989	26	0.044	0.83	26
OFT_T_groom	Cort_hair	-0.22	0.281	26	-0.35	0.0824	26
OFT_T_immob	Cort_hair	0.21	0.298	26	-0.24	0.232	26
OFT_defec	Cort_hair	0.24	0.233	26	-0.25	0.221	26
TST_LP_immob	Cort_hair	0.1	0.622	26	0.042	0.842	25
TST_N_imm	Cort_hair	0.054	0.799	25	-0.21	0.393	19
TST_T_immob	Cort_hair	0.03	0.888	25	-0.21	0.398	19
Cort_hair	Cort_hair	1	8.88E-08	26	1	8.88E-08	26
Cort_blood	Cort_hair	0.16	0.485	22	0.046	0.834	23
Thymus	Cort_hair	0.24	0.246	26	0.0065	0.975	26
Adrenals	Cort_hair	0.42	0.0345	26	-0.016	0.938	26
BW_1	Cort_hair	0.11	0.585	26	-0.3	0.138	26
BW_change	Cort_hair	-0.12	0.552	26	-0.13	0.516	26
OFT_T_centr	Cort_blood	0.21	0.34	22	0.19	0.372	23
OFT_N_centr	Cort_blood	0.28	0.211	22	0.18	0.412	23
OFT_Dist	Cort_blood	0.14	0.538	22	-0.028	0.901	23
OFT_Rear	Cort_blood	0.077	0.732	22	-0.00049	0.998	23
OFT_Holes	Cort_blood	-0.32	0.146	22	-0.13	0.564	23
OFT_T_groom	Cort_blood	-0.13	0.558	22	0.12	0.595	23
OFT_T_immob	Cort_blood	0.16	0.485	22	0.043	0.844	23
OFT_defec	Cort_blood	0.42	0.0545	22	-0.064	0.772	23
TST_LP_immob	Cort_blood	0.38	0.0793	22	-0.13	0.569	22
TST_N_imm	Cort_blood	-0.26	0.247	21	0.078	0.773	16
TST_T_immob	Cort_blood	-0.26	0.261	21	0.23	0.391	16
Cort_hair	Cort_blood	0.16	0.485	22	0.046	0.834	23
Cort_blood	Cort_blood	1	2.44E-06	22	1	1.40E-06	23
Thymus	Cort_blood	0.49	0.0193	22	0.14	0.533	23
Adrenals	Cort_blood	0.5	0.0175	22	0.09	0.681	23
BW_1	Cort_blood	0.35	0.111	22	-0.19	0.374	23
BW_change	Cort_blood	0.3	0.172	22	0.2	0.365	23
OFT_T_centr	Thymus	0.28	0.16	26	0.3	0.134	26
OFT_N_centr	Thymus	0.22	0.279	26	0.19	0.351	26
OFT_Dist	Thymus	-0.26	0.206	26	0.058	0.779	26
OFT_Rear	Thymus	-0.034	0.87	26	-0.09	0.662	26
OFT_Holes	Thymus	0.13	0.513	26	-0.096	0.64	26
OFT_T_groom	Thymus	-0.017	0.933	26	0.3	0.142	26
OFT_T_immob	Thymus	0.23	0.261	26	0.097	0.639	26
OFT_defec	Thymus	-0.044	0.832	26	0.0091	0.965	26
TST_LP_immob	Thymus	0.14	0.503	26	-0.18	0.397	25
TST_N_imm	Thymus	0.043	0.84	25	0.31	0.191	19
TST_T_immob	Thymus	-0.14	0.497	25	0.1	0.678	19
Cort_hair	Thymus	0.24	0.246	26	0.0065	0.975	26
Cort_blood	Thymus	0.49	0.0193	22	0.14	0.533	23
Thymus	Thymus	1	0	26	1	0	26
Adrenals	Thymus	0.46	0.0177	26	0.25	0.217	26
BW_1	Thymus	0.36	0.0718	26	-0.028	0.892	26

BW_change	Thymus	0.21	0.313	26	0.1	0.625	26
OFT_T_centr	Adrenals	-0.2	0.322	26	0.16	0.439	26
OFT_N_centr	Adrenals	-0.046	0.824	26	0.37	0.0667	26
OFT_Dist	Adrenals	-0.28	0.158	26	0.17	0.402	26
OFT_Rear	Adrenals	-0.3	0.132	26	0.16	0.421	26
OFT_Holes	Adrenals	-0.43	0.0283	26	0.018	0.931	26
OFT_T_groom	Adrenals	0.21	0.297	26	-0.3	0.143	26
OFT_T_immob	Adrenals	0.45	0.0223	26	-0.14	0.505	26
OFT_defec	Adrenals	0.16	0.435	26	0.43	0.0294	26
TST_LP_immob	Adrenals	0.24	0.243	26	0.22	0.286	25
TST_N_imm	Adrenals	0.0039	0.985	25	0.17	0.49	19
TST_T_immob	Adrenals	-0.18	0.397	25	-0.11	0.659	19
Cort_hair	Adrenals	0.42	0.0345	26	-0.016	0.938	26
Cort_blood	Adrenals	0.5	0.0175	22	0.09	0.681	23
Thymus	Adrenals	0.46	0.0177	26	0.25	0.217	26
Adrenals	Adrenals	1	0	26	1	0	26
BW_1	Adrenals	0.17	0.393	26	-0.039	0.851	26
BW_change	Adrenals	0.026	0.9	26	0.46	0.0175	26
OFT_T_centr	BW_1	0.22	0.273	26	-0.12	0.575	26
OFT_N_centr	BW_1	0.41	0.0387	26	0.061	0.766	26
OFT_Dist	BW_1	0.11	0.577	26	-0.074	0.718	26
OFT_Rear	BW_1	0.38	0.0527	26	-0.03	0.883	26
OFT_Holes	BW_1	-0.17	0.41	26	0.19	0.35	26
OFT_T_groom	BW_1	-0.22	0.275	26	0.3	0.135	26
OFT_T_immob	BW_1	0.19	0.361	26	-0.034	0.871	26
OFT_defec	BW_1	0.0048	0.981	26	0.16	0.449	26
TST_LP_immob	BW_1	0.59	0.00155	26	0.19	0.358	25
TST_N_imm	BW_1	-0.053	0.8	25	0.072	0.769	19
TST_T_immob	BW_1	-0.49	0.0122	25	-0.28	0.25	19
Cort_hair	BW_1	0.11	0.585	26	-0.3	0.138	26
Cort_blood	BW_1	0.35	0.111	22	-0.19	0.374	23
Thymus	BW_1	0.36	0.0718	26	-0.028	0.892	26
Adrenals	BW_1	0.17	0.393	26	-0.039	0.851	26
BW_1	BW_1	1	0	26	1	8.88E-08	26
BW_change	BW_1	-0.36	0.0716	26	-0.32	0.114	26
OFT_T_centr	BW_change	0.5	0.00851	26	0.071	0.73	26
OFT_N_centr	BW_change	0.14	0.494	26	0.22	0.286	26
OFT_Dist	BW_change	-0.21	0.298	26	0.057	0.783	26
OFT_Rear	BW_change	-0.28	0.167	26	-0.055	0.791	26
OFT_Holes	BW_change	0.13	0.543	26	0.062	0.763	26
OFT_T_groom	BW_change	-0.04	0.846	26	-0.34	0.0876	26
OFT_T_immob	BW_change	-0.098	0.634	26	-0.1	0.62	26
OFT_defec	BW_change	0.12	0.573	26	0.36	0.0719	26
TST_LP_immob	BW_change	-0.23	0.26	26	0.072	0.732	25
TST_N_imm	BW_change	0.26	0.201	25	-0.027	0.912	19
TST_T_immob	BW_change	0.56	0.00353	25	0.12	0.614	19
Cort_hair	BW_change	-0.12	0.552	26	-0.13	0.516	26
Cort_blood	BW_change	0.3	0.172	22	0.2	0.365	23

Thymus	BW_change	0.21	0.313	26	0.1	0.625	26
Adrenals	BW_change	0.026	0.9	26	0.46	0.0175	26
BW_1	BW_change	-0.36	0.0716	26	-0.32	0.114	26
BW_change	BW_change	1	0	26	1	0	26

OFT_T_centr, time in the central zone of arena; OFT_N_centr, the number of entries to the central zone of the arena; OFT_Dist, total distance travelled; OFT_Rear, the number of rearings; OFT_Holes, the number of hole pokes; OFT_T_groom, total grooming time; OFT_T_immob, total immobility time; OFT_defec, the number of defecation boluses; TST_LP_immob, latency to the first immobility episode; TST_N_immob, number of immobility episodes; TST_T_immob, total duration of immobility; Cort_hair, hair corticosterone ; Cort_blood, blood plasma corticosterone; Thymus, thymus weight ; Adrenals, adrenal glands weight ; BW_1, initial body weight; BW_change, body weight change during CUS period

Table S8. The results of MANOVA

```
=== TWO-WAY MANOVA ANALYSIS ===

Factors: group × sex
Selected variables (6): OFT_T_groom, OFT_defec, TST_LP_immob, Cort_hair, Adre
nals, BW_change


Pillai's trace test
          Df  Pillai approx F num Df den Df    Pr(>F)
group      1 0.30362   3.0520      6     42 0.0143618 *
sex        1 0.45547   5.8552      6     42 0.0001673 ***
group:sex  1 0.13430   1.0859      6     42 0.3864503
Residuals 47
---
Signif. codes:  0 ‘***’ 0.001 ‘**’ 0.01 ‘*’ 0.05 ‘.’ 0.1 ‘ ’ 1

Data quality:
  Original observations: 52
  Complete cases: 51 (98.1%)
  Variables: 6

Design balance:
  Smallest cell: 12 observations
  Largest cell: 14 observations
  Balance ratio: 0.86 (1.0 = perfect balance)
```

Variables for MANOVA were selected based on several criteria: (1) theoretical relevance to stress response domains, (2) low rates of missing data, (3) absence of profound multicollinearity, and (4) preliminary evidence of stress sensitivity in univariate analyses. The final set comprised six variables representing distinct behavioral and physiological domains: Open Field Test behaviors (n = 2), Tail Suspension Test immobility (n = 1), corticosterone levels (n = 1), and morphological measures (n = 2). This selection ensured coverage of stress-responsive systems while maintaining statistical power given sample size constraints.

Factorial multivariate analysis of variance revealed significant main effects of both experimental group (Pillai's trace = 0.304, $F(6, 42) = 3.05$, $p = 0.014$, partial $\eta^2 = 0.304$) and sex (Pillai's trace = 0.455, $F(6, 42) = 5.86$, $p < 0.001$, partial $\eta^2 = 0.455$) on the combined dependent variables. The group × sex interaction was not statistically significant (Pillai's trace = 0.134, $F(6, 42) = 1.09$, $p = 0.386$), indicating consistent treatment effects across sexes. According to Cohen's guidelines, the effect of sex was large (partial $\eta^2 \geq 0.14$), while the effect of group was medium-to-large.

Thus, in addition to univariate analyses, MANOVA provided preliminary support for both CUS and sex effects.

Table S9.

NOTE: Please save this file locally before filling in the table, DO NOT work on the file within your internet browser as changes will not be saved. Adobe Acrobat Reader (available free here) is recommended for completion.

The ARRIVE guidelines 2.0: author checklist

The ARRIVE Essential 10

These items are the basic minimum to include in a manuscript. Without this information, readers and reviewers cannot assess the reliability of the findings.

Item		Recommendation	Section/line number, or reason for not reporting
**Study design**	1	For each experiment, provide brief details of study design including:	2.2
		a. The groups being compared, including control groups. If no control group has been used, the rationale should be stated.	
		b. The experimental unit (e.g. a single animal, litter, or cage of animals).	2.2
**Sample size**	2	a. Specify the exact number of experimental units allocated to each group, and the total number in each experiment. Also indicate the total number of animals used.	2.2, 3.1-3.4
		b. Explain how the sample size was decided. Provide details of any *a priori* sample size calculation, if done.	2.2
**Inclusion and exclusion criteria**	3	a. Describe any criteria used for including and excluding animals (or experimental units) during the experiment, and data points during the analysis. Specify if these criteria were established *a priori*. If no criteria were set, state this explicitly.	2.2, Table S6
		b. For each experimental group, report any animals, experimental units or data points not included in the analysis and explain why. If there were no exclusions, state so.	3.1-3.4
		c. For each analysis, report the exact value of *n* in each experimental group.	3.1-3.4
**Randomisation**	4	a. State whether randomisation was used to allocate experimental units to control and treatment groups. If done, provide the method used to generate the randomisation sequence.	2.2
		b. Describe the strategy used to minimise potential confounders such as the order of treatments and measurements, or animal/cage location. If confounders were not controlled, state this explicitly.	2.2, Table S6
**Blinding**	5	Describe who was aware of the group allocation at the different stages of the experiment (during the allocation, the conduct of the experiment, the outcome assessment, and the data analysis).	Not applicable for stress exposure; 2.2
**Outcome measures**	6	a. Clearly define all outcome measures assessed (e.g. cell death, molecular markers, or behavioural changes).	2.2
		b. For hypothesis-testing studies, specify the primary outcome measure, i.e. the outcome measure that was used to determine the sample size.	2.2
**Statistical methods**	7	a. Provide details of the statistical methods used for each analysis, including software used.	2.7
		b. Describe any methods used to assess whether the data met the assumptions of the statistical approach, and what was done if the assumptions were not met.	2.7
**Experimental animals**	8	a. Provide species-appropriate details of the animals used, including species, strain and substrain, sex, age or developmental stage, and, if relevant, weight.	2.1
		b. Provide further relevant information on the provenance of animals, health/immune status, genetic modification status, genotype, and any previous procedures.	2.1
**Experimental procedures**	9	For each experimental group, including controls, describe the procedures in enough detail to allow others to replicate them, including:	2.1, 2.2, Table S6
		a. What was done, how it was done and what was used.	2.1, 2.2, Table S6
		b. When and how often.	2.1, 2.2, Table S6
		c. Where (including detail of any acclimatisation periods).	
		d. Why (provide rationale for procedures).	2.1, 2.2, Table S6
**Results**	10	For each experiment conducted, including independent replications, report:	3.1-3.4
		a. Summary/descriptive statistics for each experimental group, with a measure of variability where applicable (e.g. mean and SD, or median and range).	
		b. If applicable, the effect size with a confidence interval.	3.1-3.4, Supplementary

The Recommended Set

These items complement the Essential 10 and add important context to the study. Reporting the items in both sets represents best practice.

Item		Recommendation	Section/line number, or reason for not reporting
**Abstract**	11	Provide an accurate summary of the research objectives, animal species, strain and sex, key methods, principal findings, and study conclusions.	Abstract
**Background**	12	a. Include sufficient scientific background to understand the rationale and context for the study, and explain the experimental approach.	Introduction
		b. Explain how the animal species and model used address the scientific objectives and, where appropriate, the relevance to human biology.	Introduction, Discussion
**Objectives**	13	Clearly describe the research question, research objectives and, where appropriate, specific hypotheses being tested.	Introduction
**Ethical statement**	14	Provide the name of the ethical review committee or equivalent that has approved the use of animals in this study, and any relevant licence or protocol numbers (if applicable). If ethical approval was not sought or granted, provide a justification.	Institutional Review Board Statement
**Housing and husbandry**	15	Provide details of housing and husbandry conditions, including any environmental enrichment.	2.1
**Animal care and monitoring**	16	a. Describe any interventions or steps taken in the experimental protocols to reduce pain, suffering and distress.	Table S6
		b. Report any expected or unexpected adverse events.	Table S6
		c. Describe the humane endpoints established for the study, the signs that were monitored and the frequency of monitoring. If the study did not have humane endpoints, state this.	Table S6
**Interpretation/ scientific implications**	17	a. Interpret the results, taking into account the study objectives and hypotheses, current theory and other relevant studies in the literature.	Discussion
		b. Comment on the study limitations including potential sources of bias, limitations of the animal model, and imprecision associated with the results.	
**Generalisability/ translation**	18	Comment on whether, and how, the findings of this study are likely to generalise to other species or experimental conditions, including any relevance to human biology (where appropriate).	Discussion
**Protocol registration**	19	Provide a statement indicating whether a protocol (including the research question, key design features, and analysis plan) was prepared before the study, and if and where this protocol was registered.	Institutional Review Board Statement
**Data access**	20	Provide a statement describing if and where study data are available.	Data availability dtatement
**Declaration of interests**	21	a. Declare any potential conflicts of interest, including financial and non-financial. If none exist, this should be stated.	Conflicts of interest
		b. List all funding sources (including grant identifier) and the role of the funder(s) in the design, analysis and reporting of the study.	Funding
